# Caregiver and Clinician Priorities for User-Centered Metaverse-Based Speech–Language Therapy: An Analytic Hierarchy Process Study

**DOI:** 10.3390/children13101348

**Published:** 2026-10-04

**Authors:** Gyung Hun Hong, Ha-Rim Kim, Jeong-Eun Kim, Jin-Soo Lee

**Affiliations:** 1 Department of Communication Disorders, Korea Nazarene University, 48 Wolbong-ro, Seobuk-gu, Cheonan 31172, Republic of Korea; 2 Department of Communication Disorders, The Graduate School, Korea Nazarene University, 48 Wolbong-ro, Seobuk-gu, Cheonan 31172, Republic of Korea

**Keywords:** metaverse, virtual reality, speech–language therapy, children, caregivers, speech–language pathologists, analytic hierarchy process, user-centered design, needs assessment

## Abstract

**Highlights:**

**What are the main findings?**

- Caregivers and speech–language pathologists shared a strongly child-centered priority structure for metaverse-based therapy, agreeing on 9 of the 10 most important factors and diverging mainly over intervention content (caregivers prioritized content quality; clinicians prioritized content quantity and diversity).
- Among caregivers, the priority structure was stable across the child’s developmental stages: all three age subgroups produced the same domain ordering and the same child-centered leading factors. Within that stable structure, emphasis differed in degree between the younger and school-age subgroups.

**What are the implications of the main findings?**

- Because caregivers and clinicians share a developmentally grounded, child-centered core, a single therapy service may be able to address both groups’ priorities rather than requiring separate products. This is a design hypothesis consistent with the observed rankings rather than a finding established by this study.
- Developmentally staged content, role-differentiated support for caregivers and clinicians, and safety as a default should guide both clinical implementation and the design of metaverse-based pediatric speech–language therapy.

**Abstract:**

**Background/Objectives:** Metaverse technologies are increasingly considered for pediatric speech–language therapy, yet adoption has outpaced evidence on what caregivers and speech–language pathologists (SLPs)—the groups whose decisions drive uptake—consider important. This study identified and compared the priorities caregivers and SLPs assign to factors relevant to metaverse-based speech–language therapy for children, and examined whether caregiver priorities differ by developmental stage. **Methods:** In this cross-sectional formative needs assessment, a three-stage instrument-development sequence using the Analytic Hierarchy Process (AHP) produced a hierarchy of 3 high-level categories (Subject, System, Content), 8 medium-level categories, and 24 sub-factors. Caregivers of children aged 0–12 years with language development deficits, all receiving therapy (48.8% without a formal disability diagnosis, 43.2% with developmental language disorder), and practicing SLPs in Korea were recruited by convenience sampling. After data cleaning, 250 caregivers and 67 SLPs were analyzed, with caregiver data stratified into early-childhood, pre-school, and school-age groups. **Results:** Both groups ranked the high-level categories identically—Subject (67.8% vs. 67.4%), Content (18.7% vs. 18.1%), System (13.4% vs. 14.5%)—with differences ≤ 1.1 percentage points, and 9 of the 10 highest-ranked sub-factors were shared. Divergence was concentrated in Content: caregivers weighted Quality over Amount (54.7% vs. 45.3%), whereas SLPs reversed this (66.9% vs. 33.1%). Among caregivers, priorities were stable in rank order across ages, with caregiver-related factors declining and SLP-related and content-quality factors rising with age. **Conclusions:** Caregivers and SLPs converged on a strongly child-centered, developmentally grounded set of priorities while differing in content expectations. This shared structure indicates that developmentally appropriate, safety-first services may be able to address both groups’ priorities through age-adaptive content and role-differentiated support. As the sample was non-probabilistic and analyses descriptive, findings are hypothesis-generating.

## 1. Introduction

The term metaverse refers to a virtual world that enables social interactions similar to those in real-world experiences, allowing users to engage freely in virtual spaces [1]. In the present study, the metaverse is operationally defined as an immersive, persistent, and interactive virtual environment in which children, caregivers, and speech–language pathologists (SLPs) engage in therapeutic activities and communicate with one another in real time. This platform can be implemented through a range of extended reality approaches, from virtual reality (VR) and augmented reality (AR) to mirror worlds and lifelogging.

As a medium that offers novel experiences within spaces integrating the real and the virtual, the metaverse has drawn increasing attention as a promising platform for future education because of its potential to support immersive, interactive, and collaborative learning experiences [2,3,4]. Metaverse-based learning environments have been reported to enhance learner interaction and support the resolution of real-world problems [4,5]. Similarly, immersive technologies such as VR and AR can foster engagement and interactivity, making them particularly effective in applied educational and rehabilitation contexts [6,7].

This uptake has accelerated sharply since the COVID-19 pandemic, as demand grew for real-world-equivalent experiences in online environments [8]. Reported benefits of VR- and AR-based intervention in speech–language therapy span the acquisition of question forms in children with autism spectrum disorder (ASD), vocabulary acquisition, sentence formulation, and emotional expression [9,10,11,12,13].

Yet this momentum has outpaced the evidence needed to deploy the technology safely with children. Several important limitations have been documented. Children with intellectual disabilities or ASD may require individualized preparation and ongoing monitoring during VR-based interventions to support safe and effective participation [14]. The clinical utility of virtual programs is frequently constrained by hardware liabilities—such as high costs, inappropriate software sizing, and excessive device weight—in addition to user-specific barriers, including an intolerance of wearing headsets or glasses [15]. Concurrently, immersive VR programs for young children have raised important concerns regarding safety and potential adverse side effects [16]. When real-world adoption advances faster than an understanding of what makes a metaverse-based intervention developmentally appropriate, clinically feasible, and safe, the field risks building platforms that fail the very users they target. Closing that risk requires evidence on what the people responsible for therapy actually prioritize.

Caregivers play a critical role in children’s language development and are active participants in intervention [17,18], while SLPs set and implement therapy goals and would be the primary practitioners deploying metaverse technologies in clinical settings. Their perceptions and attitudes therefore largely determine whether metaverse-based speech–language therapy is adopted, adhered to, and sustained. To date, however, most studies have focused on the effectiveness of technology-mediated speech–language interventions, with relatively little attention paid to the factors that caregivers and SLPs consider important for adoption and sustained use. This gap is significant because stakeholder priorities influence not only initial acceptance but also long-term engagement and implementation. Furthermore, caregivers and SLPs may value different features of metaverse-based services; without evidence regarding these priorities, the design of future platforms is likely to rely on assumptions rather than user needs.

This issue is particularly relevant for metaverse-based services, which differ from conventional digital interventions by integrating immersive interaction, persistent virtual spaces, and real-time social participation. As a result, factors related to safety, privacy, environmental control, content characteristics, and social participation may play an important role in stakeholder acceptance and continued use.

Given the limited evidence regarding stakeholder expectations for metaverse-based speech–language therapy, this study was designed as a formative needs-assessment study prior to platform development. Identifying stakeholder priorities before system development is a core principle of user-centered design and has been recommended in eHealth development frameworks, which position stakeholder involvement from the earliest stage as a determinant of subsequent uptake and impact [19].

The present study addresses this gap by using the Analytic Hierarchy Process (AHP), a structured multi-criteria method that derives the relative importance of competing criteria from pairwise comparisons [20]. AHP was selected because it decomposes a complex judgment into tractable comparisons, yields quantitative weights that can be compared across stakeholder groups, and provides an internal check on judgment consistency; it has been applied extensively in healthcare decision-making and priority-elicitation research [21]. Specifically, this study identifies and compares the priorities that caregivers and SLPs in Korea assign to factors relevant to the application of metaverse technologies in children’s speech–language therapy, and examines how caregiver priorities vary according to the child’s age. The findings are intended to inform the ethical, developmentally appropriate, and user-centered design of future metaverse-based speech–language therapy platforms and services.

The research questions were as follows:

What differences exist between caregivers and SLPs in the priorities they assign to factors relevant to the application of the metaverse in speech–language therapy?

How do priority rankings for these factors differ among caregiver subgroups according to the age range of the child?

## 2. Materials and Methods

### 2.1. Study Design

**Figure 1:**
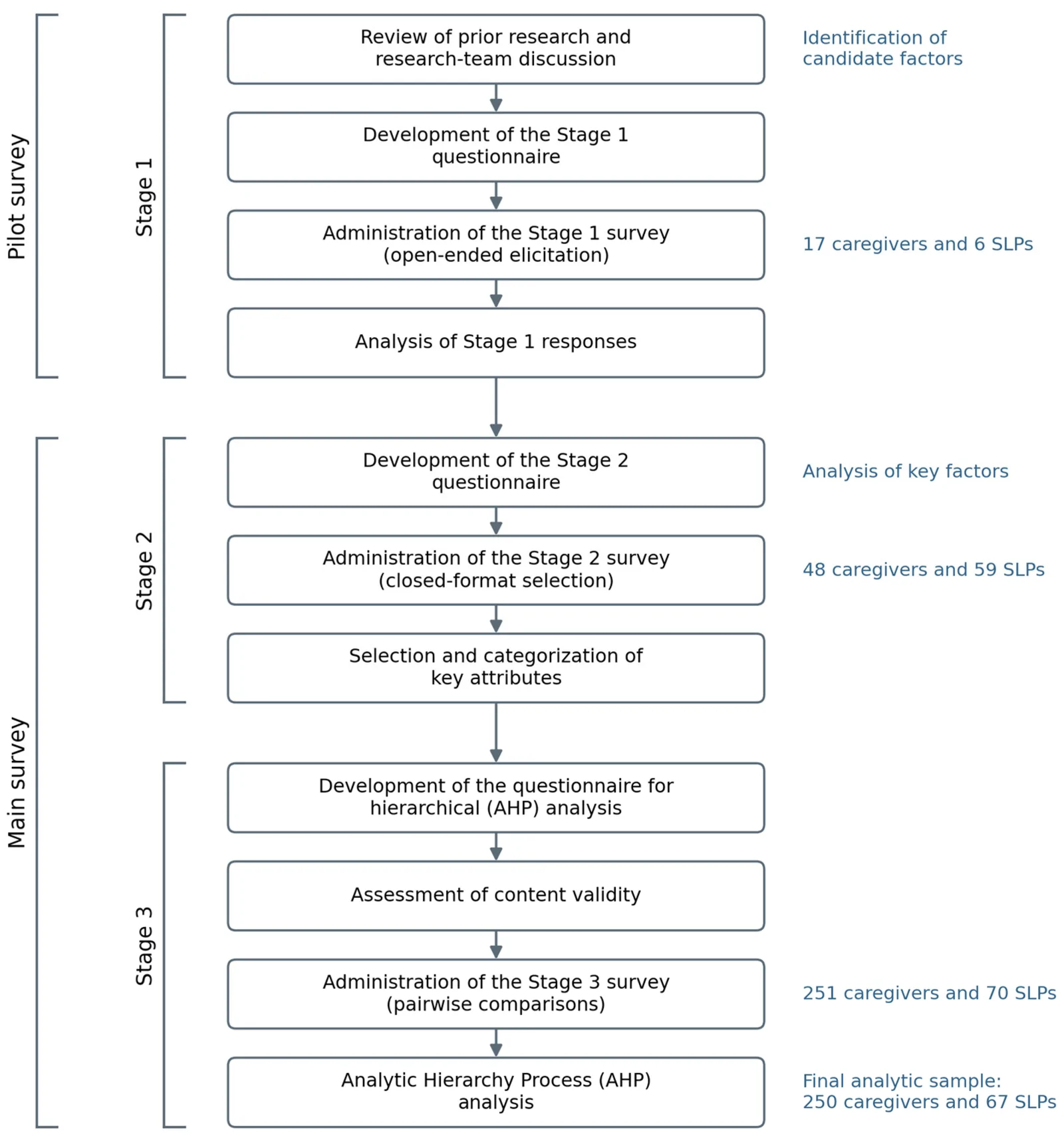
Research procedure. Note. AHP = Analytic Hierarchy Process; SLP = speech–language pathologist. At Stage 3 the platform enforced a consistency ratio (CR ≤ 0.20) in real time, so 251 caregivers and 70 SLPs submitted complete, consistent responses; the final analytic sample (250 caregivers, 67 SLPs) reflects the removal of four records with incomplete eligibility or classification items, itemized in Section 2.5. Each stage supplied the input for the next: Stage 1 generated candidate considerations through open-ended elicitation, Stage 2 narrowed them to a closed set of key attributes, and Stage 3 organized the retained attributes into the final hierarchy and elicited pairwise comparisons.

This study employed a cross-sectional formative needs-assessment design using the AHP. Data were collected in three sequential stages, each supplying the input for the next: a pilot survey using open-ended elicitation (Stage 1), a closed-format selection survey (Stage 2), and the main AHP pairwise-comparison survey (Stage 3). Stages 2 and 3 together constituted the main survey. The stage labels Stage 1, Stage 2, and Stage 3 are used consistently throughout this report. Figure 1 summarizes the full procedure.

### 2.2. Ethical Considerations

The study was determined to be exempt from full ethical review by the Institutional Review Board of Korea Nazarene University (approval code 2024-Sinsok-10; date of approval: 4 June 2024); the formal statement appears at the end of the article. The exemption was applied for on 9 April 2024, resubmitted with revisions on 8 May 2024, and granted on 4 June 2024. The Board has since confirmed in writing that the determination rests on the nature of the research—an anonymous, non-interventional survey collecting no personally identifiable information and posing no more than minimal risk—and therefore applies to the study as a whole, including the two preliminary stages, irrespective of when each stage was conducted; the confirmation has been provided to the editorial office. All participants were informed of the study purpose and provided electronic informed consent before responding. Respondents who scanned the QR code or followed the link were taken first to a consent page describing the study, the voluntary nature of participation, and the compensation offered; the survey items became accessible only after the mandatory consent item had been selected. Participation was voluntary, and responses were collected without direct identifiers. At the end of the survey, respondents could consent to provide contact details to receive a mobile coffee voucher through a social networking service; these details were used solely to deliver the voucher, were not linked to the survey responses in the analysis dataset, and were deleted once the vouchers had been issued.

### 2.3. Participants

Participants were caregivers of children aged 0–12 years and SLPs residing across Korea, recruited by convenience sampling. All children in the caregiver sample had a language development deficit and were attending a speech–language therapy service at the time of the survey; a formal disability diagnosis was not required for eligibility. Eligibility was defined functionally rather than diagnostically because a substantial proportion of children receiving speech–language therapy in Korea do not hold a formal disability diagnosis; requiring one would have excluded almost half of the actual service-using population and would have biased the priorities that this user-centered study set out to capture. Diagnoses were reported by caregivers and were not clinically verified, and the item asked for a single primary diagnosis, so the categories are mutually exclusive and comorbid conditions were not captured. The questionnaire did not ask whether a formal diagnostic evaluation had been conducted or recommended at enrolment. Within the analytic caregiver sample, just under half of the children had no disability diagnosis (*n* = 122, 48.8%), and the remainder had a range of diagnoses, the most common being developmental language disorder (DLD; *n* = 108, 43.2%), followed by intellectual disability (*n* = 14, 5.6%), autism spectrum disorder (ASD; *n* = 5, 2.0%), and speech sound disorder (*n* = 1, 0.4%); the full distribution is given in Table S1. For analysis, children were classified into three age groups: early childhood (0–3 years), pre-school (4–6 years), and school-age (7–12 years). Stage 1 involved 17 caregivers and 6 SLPs. Stage 2 was completed by 48 caregivers and 59 SLPs (*N* = 107); within the Stage 2 caregiver sample, children were distributed as early childhood (*n* = 20), pre-school (*n* = 11), and school-age (*n* = 17). Recruitment targets were set in advance in the approved study protocol (25 participants for Stage 1, 75 for Stage 2, and 550 for Stage 3). Because AHP derives relative priorities from the internal consistency of individual judgments rather than from population sampling, a conventional power calculation does not apply; the targets followed established practice for stepwise AHP instrument development. The stability of the resulting estimates was examined empirically and is reported in Section 3.1.

Eligibility as an SLP rested on recruitment through speech–language therapy institutions and on self-report of current clinical practice; the questionnaire recorded years of clinical experience but did not include an item verifying the grade of the national speech–language pathologist certificate, so the certification profile of the sample cannot be described. This is stated as a limitation. At Stage 3, 251 caregivers and 70 SLPs submitted responses. The SSRA platform computed the CR for each set of pairwise comparisons the moment a respondent pressed “next”: if consistency fell below the researcher-defined criterion (CR ≤ 0.20 in this study), the screen displayed a prompt asking the respondent to re-examine their answers, and the page would not advance until the comparisons were revised. Because a response could not be submitted unless it satisfied the CR criterion, essentially no respondents were excluded on consistency grounds. Four records were removed because an eligibility or classification item was missing; the reasons are itemized in Section 2.5. This left a final analytic sample of 250 caregivers and 67 SLPs. Full demographic characteristics of both groups are provided in Table S1. Within this analytic sample, two different caregivers each omitted a single demographic item, which was retained; because neither item was used in the AHP computations or in the age-based stratification, this had no effect on the analyses.

### 2.4. Questionnaire Development

The questionnaire was developed across three sequential stages—open-ended elicitation (Stage 1), closed-format selection (Stage 2), and the final AHP pairwise-comparison survey (Stage 3)—each supplying the input for the next. The sequence was designed to ground the AHP hierarchy in stakeholder-informed content rather than in categories imposed at the outset by the research team. The final attributes were nevertheless selected by triangulating stakeholder responses with the literature reviewed at Stage 1, the researchers’ clinical experience, and research-team discussion, so the hierarchy reflects investigator input as well as participant input. Because all three surveys were administered anonymously and no identifiers were collected, it cannot be determined whether any individual participated in more than one stage.

The coding and selection procedures that produced the hierarchy were as follows. At Stage 1, each of the three researchers independently extracted the considerations contained in every open-ended response before the extracted items were compared item by item. Because this was an attribute-generation step from open text rather than classification into a predefined frame, no chance-corrected agreement coefficient was computed and the procedure was consensus-based; this is a limitation of the audit trail. The independently extracted items were merged into a single coding sheet under four rules: organization into a category–subcategory structure by question domain and respondent group; naming aligned to the terminology used in the telepractice and metaverse literature; provisional classification of unclear items as “other”, later absorbed into an existing category or made into a new one after discussion; and a check of the resulting scheme against the ASHA Telepractice Practice Portal categories and the Facilitator Checklist for Telepractice, so that clinically necessary considerations rarely mentioned by respondents were not lost. This process organized the extracted items into 91 named subcategories across the 12 elicitation items. At Stage 2, selection frequencies were computed for each option, and options were presented in randomized order using the survey platform’s option-randomization setting to reduce order effects. The rules for retaining attributes at Stage 2 and for constraining the size of the hierarchy are described in the paragraphs that follow. The mapping from Stage 1 concepts through Stage 2 options to the 24 sub-factors, with selection rates, is provided as Supplementary Table S5.

Before responding at Stages 1 and 2, participants received standardized explanatory materials on the metaverse: an operational definition, and a comparison table of the four metaverse types (augmented reality, lifelogging, mirror worlds, and virtual worlds) specifying each type’s definition, value proposition, representative commercial services, and known drawbacks, with illustrative images, adapted from a national technology-policy report. Participants were then informed of the study purpose and provided electronic informed consent.

Stage 1: Open-ended elicitation. A review of prior research on metaverse applications in education and speech–language pathology, combined with research-team discussion, identified four domains for elicitation: (a) the rationale for using the metaverse in speech–language therapy, (b) the system requirements it imposes, (c) the problems it might create, and (d) how it differs from conventional face-to-face therapy. Domains (a), (c), and (d) were each elicited separately with respect to the child, the caregiver, and the SLP; domain (b) was elicited as a single item. Every item was accompanied by one worked example to calibrate the expected level of specificity (e.g., “eliciting the child’s interest”), and respondents were asked to list as many considerations as possible. Stage 1 additionally recorded participants’ self-rated familiarity with the metaverse (5-point scale) and prior metaverse experience—their own and their child’s—including the types experienced.

Stage 2: Closed-format selection. Three researchers independently content-analyzed the Stage 1 responses. Candidate attributes were derived by triangulating three sources: the frequency with which each consideration was raised, the researchers’ clinical experience, and the literature reviewed at Stage 1. This triangulation was adopted because spontaneous mention frequency reflects salience rather than importance, and attributes that are clinically essential but rarely articulated—data protection and network reliability among them—would otherwise have been lost. Discrepancies among the three analysts were resolved by discussion until consensus.

The procedure yielded 13 categories organized under three headings: (a) considerations by participant (child, caregiver, SLP; 3 categories); (b) considerations by metaverse component (device/system/physical environment, content, child, caregiver, SLP; 5 categories); and (c) advantages relative to conventional therapy, elicited with respect to the same five facets used under heading (b)—device/system/physical environment, content, child, caregiver, and SLP (5 categories). Each category was presented with its six highest-ranked attributes, producing 78 items, and respondents selected the three to four attributes they considered most important within each. Each of the three sections closed with a free-text item inviting any consideration not covered by the listed options, providing a check on the completeness of the attribute pool.

Stage 3: Hierarchy construction and AHP survey. The attributes with the highest selection rates at Stage 2 were carried forward and organized into a three-level hierarchy through research-team discussion, retaining the three sub-factors with the highest selection rates within each emerging medium-level category so that every cluster held two or three elements. The heading “advantages relative to conventional therapy” was not retained as a separate branch: Stage 2 responses indicated that these attributes expressed perceptions about the users, the system’s functions, and the characteristics of the content rather than a distinct construct, and they were therefore redistributed across the remaining branches. The heading “metaverse components” was divided into System and Content to yield a clearer decomposition, producing three high-level categories: Subject, System, and Content.

**Figure 2:**
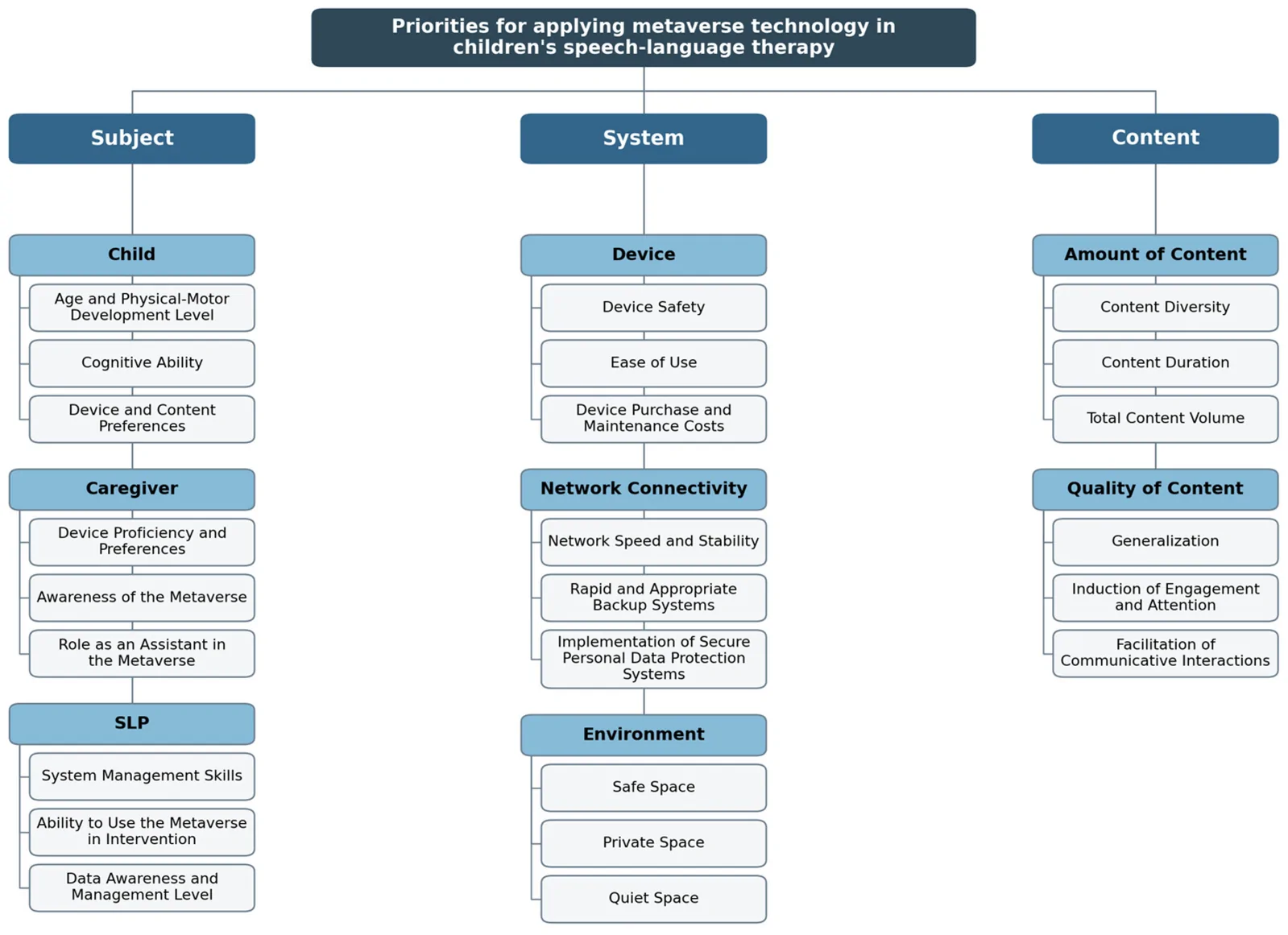
Hierarchy of categories and sub-factors used in the Analytic Hierarchy Process survey. Note. SLP = speech–language pathologist. The final hierarchy comprised 3 high-level categories, 8 medium-level categories, and 24 sub-factors, requiring 34 pairwise comparisons in total. Each cluster was capped at two or three elements to limit respondent burden and inconsistency.

The size of the hierarchy was constrained deliberately. Because the number of pairwise comparisons within a cluster grows as n(n − 1)/2, and because long comparison batteries depress both completion rates and response consistency [20,21], each cluster was capped at two or three elements. This constraint reflects the established limit on the number of items that can be held in mind and judged reliably at once [22], which Saaty and Ozdemir formalized for the AHP by showing that consistency deteriorates as the number of elements within a cluster rises [23]. The final structure comprised 3 high-level categories, 8 medium-level categories (two or three per high-level category), and 24 sub-factors (three per medium-level category), requiring 34 pairwise comparisons in total (Figure 2).

**Figure 3:**
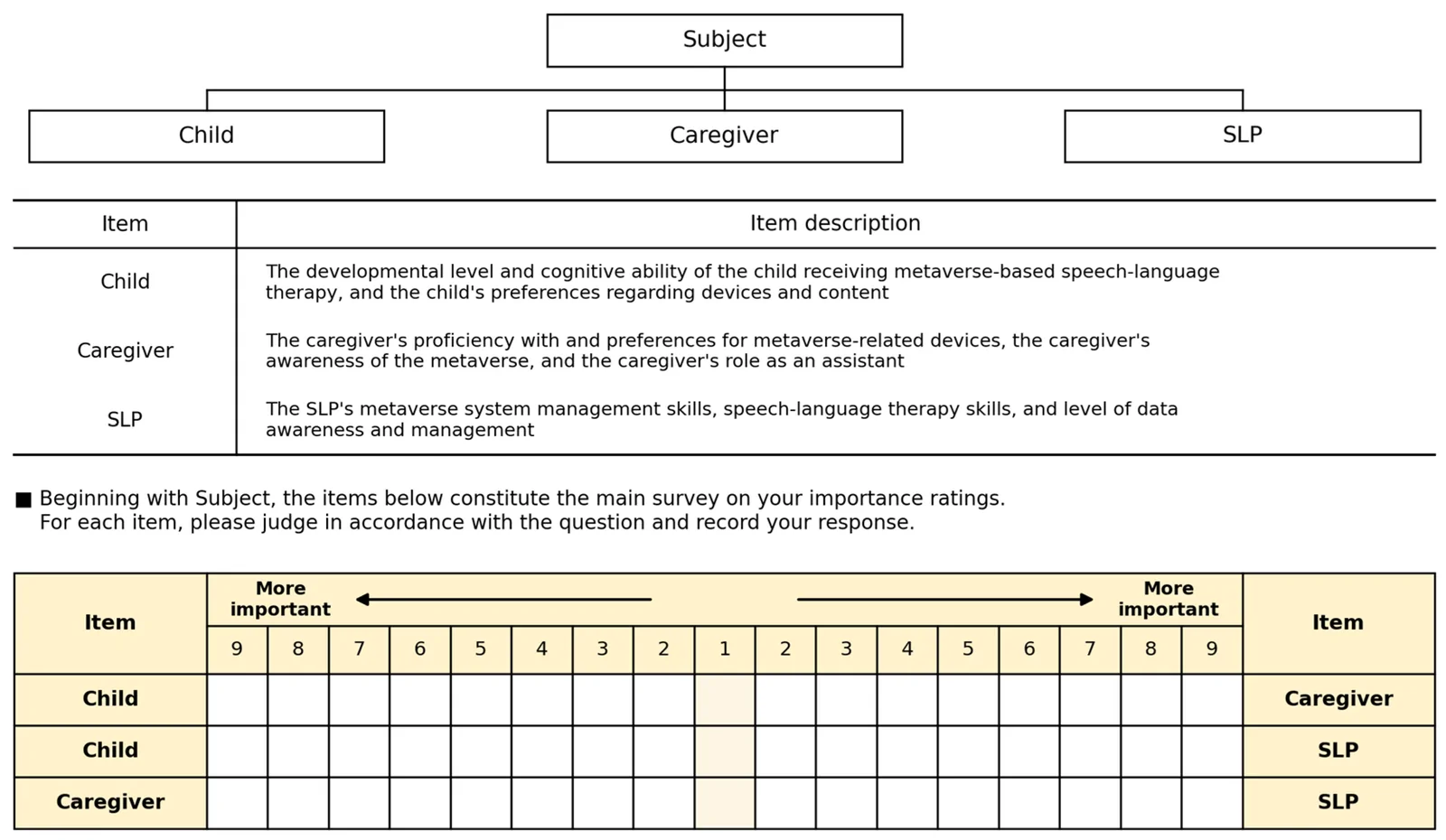
Sample item from the Analytic Hierarchy Process questionnaire (Subject cluster), translated from the Korean original. Note. SLP = speech–language pathologist. Excerpt from the Stage 3 instrument. Each cluster of the hierarchy was presented in this format: a partial hierarchy identifying the elements to be compared, a table giving the operational definition of every element in the cluster, and the pairwise-comparison items. Respondents marked one cell per row on Saaty’s 9-point ratio scale, where 1 denotes equal importance and 9 denotes that the element on the corresponding side is absolutely more important. The Subject cluster shown here required 3 pairwise comparisons. The double-headed arrow indicates the direction of relative importance: a mark closer to either end denotes greater importance of the element on that side.

Judgments were elicited on Saaty’s 9-point ratio scale, anchored at “equally important (1)” and “absolutely more important (9)” [20]. The instrument opened with an explanation of the pairwise-comparison logic, a worked example of a completed comparison, and a description of the consistency criterion together with the two ways inconsistency arises (violations of ordinal transitivity and of cardinal proportionality). Each cluster was then presented in a standard three-part format: a partial hierarchy identifying the elements to be compared, a table giving the operational definition of every element in that cluster, and the pairwise-comparison items themselves. Respondents were instructed to read the definitions carefully before making any comparison. Figure 3 reproduces the Subject cluster as an illustrative example. Content validity of the sub-factor definitions was assessed by four SLPs on a 5-point scale. Twenty-three of the 24 sub-factors were rated; the definition of Device Proficiency and Preferences was inadvertently omitted from the review, which is noted as a limitation. The mean rating was 4.61 (item means 4.25–5.00), and the item-level content validity index, computed as the proportion of experts rating an item 4 or above, ranged from 0.75 to 1.00 with a scale-level index (S-CVI/Ave) of 0.891. The prespecified criteria for retention were an item mean of 4.00 or above and an I-CVI of 0.75 or above; every rated item met both, so no item was deleted and the structure of the hierarchy was unchanged. One definition was clarified in response to the review: examples of devices were added to the definition of Device and Content Preferences. Item-level ratings and indices are provided as Supplementary Table S4.

The AHP instrument was identical for both groups—the same 12 clusters, 24 sub-factors, operational definitions, item order, and scale anchors—differing only in the demographic section. Between-group comparisons are therefore not confounded by instrument differences.

### 2.5. Data Collection

All data were collected in 2024: Stage 1 from 23 to 30 April, Stage 2 from 21 May to 4 June, and Stage 3 from 18 to 30 June. The Stage 1 and Stage 2 questionnaires were built in Google Forms (Google LLC, Mountain View, CA, USA) and distributed by URL and QR code. For Stage 1, researchers contacted caregivers and SLPs individually. For Stage 2, recruitment was extended by approaching institutions providing speech–language services; the questionnaire was distributed to SLPs working at those institutions and to caregivers using them. Both stages required responses to all items before submission.

The same standardized explanatory material on the metaverse was presented at Stages 1 and 2, and the Stage 3 questionnaire opened with an equivalent introduction, so participants at every stage judged the same described system. The Stage 3 explanatory material and the full Stage 3 questionnaire are provided in the Supplementary Materials in the Korean original with an English translation.

The Stage 3 questionnaire was built using the Social Science Research Automation System (SSRA; www.ssra.or.kr; a non-commercial academic platform maintained in the Republic of Korea), a cloud platform for automating social-science research. Recruitment for Stage 3 extended the pool of Stage 2 participants by approaching a larger number of speech–language therapy institutions. Each institution was given a promotional recruitment poster that stated the purpose of the study, a brief description of the survey, and the compensation offered, and that carried the survey link as a QR code. The URL itself was sent to the institutional contact rather than printed on the poster. Participating institutions displayed the poster on-site, and their SLPs and the caregivers who used them took part voluntarily by scanning the code; the survey was also distributed through social networking services (SNS). The platform enforced consistency in real time: after a respondent completed a set of pairwise comparisons and pressed “next,” it immediately computed the CR for that set.

Because the survey platform submitted a response only when every pairwise comparison had been completed, the analytic dataset contained no missing judgment values (0 of 8500 cells for caregivers and 0 of 2278 for SLPs). Exclusion therefore rested on the complete-response rule for the 34 pairwise comparisons together with completeness of the eligibility and classification items, and no proportional threshold for missing data was applied. On this basis one caregiver record was excluded for a missing child age-band item, and three SLP records were excluded, two for a missing item on the age band of children usually seen and one for a missing item on the disability types usually seen. Compensation was a mobile coffee voucher worth KRW 2000, issued after completion to the telephone number respondents supplied for that purpose; vouchers were sent to the 190 respondents across Stages 2 and 3 who consented to provide a contact number. Vouchers were funded from the research grant and were therefore subject to its disbursement rules, under which a given telephone number may receive payment only once; compliance was checked by the research team and by the institution’s administrative office. This provided a limited check against repeat submission among respondents who supplied a contact number, although it would not have detected the same individual using different numbers and did not apply to those who declined to provide one. No platform-level control against duplicate submission was applied: restricting the form to one response per respondent would have required participants to sign in to a Google account and would have linked responses to identifiable accounts, which was incompatible with the anonymous administration on which the ethics waiver rested, and the platform does not make respondent IP addresses available. Anonymity was therefore prioritized over technical duplicate prevention, and the consequent inability to detect repeat submission is acknowledged in the Limitations section.

Across the main survey (Stages 2 and 3), distribution was concentrated in the Incheon, Gyeonggi, and Chungcheong regions, with additional responses obtained from Seoul and parts of the Gyeongsang region. Recruitment was therefore neither nationally representative nor probability-based.

### 2.6. Data Analysis

The AHP is a multi-attribute decision-making method that hierarchically classifies attributes and derives relative priorities through stepwise pairwise comparison [20,24,25]. Its defining feature is the derivation of importance weights from pairwise comparisons on a 9-point ratio scale. Data were collected via the SSRA platform and analyzed using DRESS software (Version 1.7.00; CHOISH Software, Seoul, Republic of Korea). Individual pairwise judgments were aggregated before priorities were derived (aggregation of individual judgments, AIJ). Each off-diagonal element of the aggregated matrix was the geometric mean of all respondents’ judgments for that cell, $a_{\mathrm{ij}}={\;\left(\prod_{k}a_{\mathrm{ij}}^{\left(k\right)}\right)}^{\frac{1}{n}}$, with reciprocal elements a_ji = 1/a_ij; the geometric mean is the only aggregation function that preserves reciprocity of the aggregated matrix. All respondents were weighted equally (1/n), with no differential or stratified weighting, and the two groups were aggregated independently. Priority vectors were obtained by the principal eigenvector method and normalized to sum to one; for the reciprocal matrices of order two and three used here, the principal-eigenvector solution coincides with the row geometric-mean (logarithmic least squares) solution, so either method reproduces the same weights. Global sub-factor weights were obtained by distributive synthesis, that is, the product of the high-level weight, the medium-level local weight, and the sub-factor local weight. A sensitivity check confirmed that AIP aggregation with the geometric mean reproduced these weights exactly. The aggregated pairwise-comparison matrices for all 12 clusters, with their eigenvalues, consistency indices and derived weights, are provided as Supplementary Table S2.

To verify the logical consistency of respondents’ judgments, the consistency ratio (CR) proposed by Saaty was computed [20]. The CR expresses the consistency index CI = (λmax − n)/(n − 1) relative to the index expected from randomly generated matrices of the same order, using RI = 0.58 for n = 3; AHP studies conventionally adopt CR < 0.10 [20,26]. Of the 12 comparison matrices completed by each respondent, 11 were of order three and one—the Content cluster—was of order two; a reciprocal 2 × 2 matrix is consistent by construction, so CR carries no discriminative information there and the real-time check operated on the 11 third-order matrices.

This study pre-specified the more permissive criterion of CR ≤ 0.20, which the SSRA platform applied in real time as described above. The printed instruction sheet, reproduced in the Supplementary Materials, states the conventional criterion of 0.10; the threshold implemented in the platform was 0.20, and the distribution of recomputed CR values confirms that this was the criterion in force. That threshold is appropriate to the present setting: each respondent completed 34 pairwise comparisons whose criteria became progressively finer at lower levels of the hierarchy, and respondents were caregivers and practising clinicians completing the task online rather than trained decision analysts, conditions under which minor cognitive error accumulates. The same criterion has been pre-specified in health-informatics AHP studies eliciting priorities from patients and other non-expert respondents [27,28], and a direct comparison of the two thresholds in that setting found that mean weights derived under CR ≤ 0.10 and under CR ≤ 0.20 did not differ significantly [29]. Because the platform required every comparison set to satisfy this criterion before a response could be submitted, essentially no records were excluded on consistency grounds; the small reduction from the submitted to the analytic sample reflected the removal of records with missing eligibility or classification items.

The platform enforced the criterion at entry rather than logging each respondent’s value, but because the individual judgments were retained, CR was recomputed post hoc for every respondent. Taking each respondent’s least consistent matrix, the median maximum CR was 0.046 among caregivers and 0.117 among SLPs; all 250 caregivers and all 67 SLPs satisfied CR ≤ 0.20, and 167 caregivers (66.8%) and 32 SLPs (47.8%) also satisfied the stricter CR ≤ 0.10. A sensitivity analysis restricted to those respondents reproduced the reported ordering closely (Spearman ρ = 0.976 for caregivers and 0.964 for SLPs against the full-sample weights), with the same five highest-ranked sub-factors among caregivers and four of five among SLPs, so the more permissive threshold did not drive the findings.

AHP analyses were conducted separately for the caregiver and SLP groups, with caregiver data additionally stratified into the three age-based subgroups (early childhood, pre-school, and school-age). Sub-factor weights are reported as global weights, that is, the product of the weights along each branch of the hierarchy; within each group they sum to 1.00 across the 24 sub-factors. Priorities are reported as weights and ranks, and the inferential comparisons described below are reported alongside them as supporting evidence.

Because aggregate weights alone convey no information about dispersion or agreement, individual-level priority vectors were reconstructed for every respondent and used for three further analyses. Dispersion is reported as the median and interquartile range of the individual weights. Two distinct bootstrap procedures were used, and they estimate different quantities. For the aggregate weights reported in Table 1, Table 2 and Table 3, respondents were resampled with replacement within each group (2000 resamples) and the full aggregation pipeline—geometric-mean aggregation of the individual judgments, extraction of the principal eigenvector, and distributive synthesis—was re-run on every resample, so that the resulting percentile intervals propagate uncertainty through every step; these intervals are given in Supplementary Table S6. For the comparison between groups, the individual-level weights themselves were resampled to obtain intervals for the difference in means; these are the intervals reported in Supplementary Table S3. The analysis script for both procedures is available from the corresponding author on reasonable request. Within-group agreement on the ranking of the 24 sub-factors was quantified with Kendall’s coefficient of concordance, and between-group agreement with Spearman’s rank correlation. Group differences were tested with Mann–Whitney U tests applied to the individual weights, with rank-biserial correlation as the effect size and Benjamini–Hochberg correction applied separately within the high-level (3 tests), medium-level (8 tests), and sub-factor (24 tests) families. Because weights within any cluster are constrained to sum to one, these tests are applied to compositional data and the comparisons within a cluster are not independent; they are therefore reported as supporting evidence alongside the interval estimates rather than as independent confirmatory tests. The individual-level reconstruction and the inferential analyses reported here were carried out with the assistance of a generative AI tool (Claude, Anthropic PBC, San Francisco, CA, USA), which was used to write and execute the analysis scripts in Python 3.12.3 (numpy 2.4.4, scipy 1.17.1). The authors verified the outputs against the aggregated matrices produced independently by DRESS v1.7.00, which the reconstruction reproduced to four decimal places.

The same procedure was applied to two further comparisons within the caregiver sample. Differences across the three age subgroups were tested with Kruskal–Wallis tests, with epsilon squared as the effect size. Priorities were also derived separately for children with developmental language disorder (n = 108) and children without a diagnosis (n = 122) and compared with Mann–Whitney U tests; the remaining diagnostic categories—intellectual disability (n = 14), autism spectrum disorder (n = 5) and speech sound disorder (n = 1)—were too small to yield stable weights and were not analyzed separately.

## 3. Results

### 3.1. Priorities by Caregiver and SLP Groups

#### 3.1.1. High-Level Categories

Both groups ranked the high-level categories identically: Subject (rank 1), Content (rank 2), and System (rank 3). The two groups were also closely matched in the magnitude of these weights. Subject accounted for approximately two-thirds of the total weight in both groups (caregivers 67.8%; SLPs 67.4%), more than twice the combined weight of Content (18.7% and 18.1%) and System (13.4% and 14.5%). Between-group differences did not exceed 1.1 percentage points for any of the three categories. At the highest level of the hierarchy, therefore, caregivers and SLPs were closely aligned: both located the decisive considerations in the people involved in therapy rather than in the content or the technology (Table 1).

#### 3.1.2. Medium-Level Categories

The two groups agreed at the medium level in two of the three domains and diverged in the third. Within Subject, both ranked Child first by a wide margin (caregivers 63.1%; SLPs 64.3%), followed by SLP (19.9% and 20.9%) and then Caregiver (16.9% and 14.8%). Within System, both ranked Device first (61.8% and 64.6%), followed by Environment (21.9% and 20.0%) and then Network Connectivity (16.3% and 15.4%). Between-group differences were no greater than 2.8 percentage points for any of these categories, indicating close agreement in both rank order and relative weight.

The one clear divergence occurred within Content. Caregivers weighted Quality of Content slightly above Amount of Content (54.7% vs. 45.3%), an almost even split, whereas SLPs reversed the ordering decisively, assigning about twice as much weight to Amount of Content as to Quality of Content (66.9% vs. 33.1%). At the aggregate level caregivers therefore showed little preference between the two aspects of content, with a modest tilt towards quality, whereas SLPs expressed a pronounced preference for the availability of content (Table 2).

The individual-level analyses qualify this picture in three ways. First, agreement on the ordering of the 24 sub-factors was moderate rather than high within each group (Kendall’s W = 0.43 among caregivers and 0.44 among SLPs, both *p* < 0.001), while the two aggregate rankings themselves corresponded closely (Spearman ρ = 0.92, *p* < 0.001). Second, the group difference was confined to Content. None of the three high-level comparisons reached significance after Benjamini–Hochberg correction (all q ≥ 0.43), and of the eight medium-level comparisons only the Content pair did (q = 0.039, rank-biserial r = 0.20); of the 24 sub-factor comparisons, the five that reached significance were all Content sub-factors—Content Diversity (q = 0.035), Content Duration (q = 0.012), Total Content Volume (q = 0.008), Induction of Engagement and Attention (q = 0.008), and Facilitation of Communicative Interactions (q = 0.033). Differences in Cognitive Ability (q = 0.91) and Device Safety (q = 0.41) were not statistically distinguishable, and those descriptive gaps should therefore not be read as reliable group differences. Third, the near-balance between the two Content sub-domains among caregivers is a property of the aggregate rather than of individual respondents: the distribution of individual weights for Amount of Content was strongly bimodal, with 143 caregivers placing more weight on Quality and 92 on Amount and few taking an intermediate position (median 0.20, IQR 0.17–0.86). Full test statistics for all comparisons are provided as Supplementary Table S3. Caregivers were therefore divided on this question rather than indifferent to it, and a service specification derived from the aggregate alone could misrepresent them.

Because a sub-factor’s global weight is the product of the weights of its parent domains, differences in global rank do not by themselves indicate differences in preference for that sub-factor. Local weights are therefore reported alongside global weights in Table 3. The Content sub-factors illustrate the distinction: SLPs assigned Content Diversity a higher global weight than caregivers did (0.077 versus 0.056), but the local weight of that sub-factor within Amount of Content was similar in the two groups (0.636 versus 0.663); the global difference arises almost entirely from the weight the two groups placed on Amount of Content relative to Quality of Content (0.669 versus 0.453).

#### 3.1.3. Sub-Level Factors

Across the 24 sub-level factors, both groups concentrated their top priorities on a shared set of child-, content-, and safety-related items. Because the complete rankings are presented in Table 3, only the factors that best illustrate the agreements and disagreements between the two groups are described here.

Agreement was extensive. Nine of the ten highest-ranked sub-factors were common to both groups. The child’s Age and Physical-Motor Development Level ranked first by a wide margin in both (caregivers 27.4%; SLPs 24.8%), carrying close to three times the weight of the second-ranked factor in each group and, on its own, exceeding the weight of the entire System domain. The shared top ten also comprised the child’s Device and Content Preferences (8.3% and 9.2%), the SLP’s System Management Skills (7.8% and 8.2%), the child’s Cognitive Ability, the caregiver’s Device Proficiency and Preferences (6.3% and 6.1%), Content Diversity (under Amount of Content), Generalization (under Quality of Content), Device Safety, and the SLP’s Data Awareness and Management Level (3.0% in both groups). The single non-overlapping item in each list—the caregiver’s Role as an Assistant in the Metaverse for caregivers and the SLP’s Ability to Use the Metaverse in Intervention for SLPs—carried an identical weight of 2.9% in whichever group ranked it tenth.

The two groups also agreed on what mattered least. Network Speed and Stability ranked 19th in both (1.3% and 1.2%), and the four lowest ranks were occupied by the same factors in each group—Quiet Space and Private Space (under Environment), Implementation of Secure Personal Data Protection Systems and Rapid and Appropriate Backup Systems (under Network Connectivity), each no more than 0.7%—with Rapid and Appropriate Backup Systems last in both (0.4%).

Two child- and device-related factors differed descriptively between the groups. The child’s Cognitive Ability showed the largest descriptive gap: SLPs ranked it second (9.3%), immediately after the child’s Age and Physical-Motor Development Level, whereas caregivers ranked it fourth (7.1%), behind both the child’s Device and Content Preferences and the SLP’s System Management Skills. SLPs also assigned more weight to Device Safety than caregivers did (6.0%, rank 7, vs. 4.8%, rank 8). Neither difference was statistically distinguishable after adjustment (q = 0.91 and q = 0.41 respectively), and both are therefore reported as descriptive differences only.

The most systematic divergence concerned Content, where the sub-level results reproduced the medium-level reversal without exception. SLPs weighted every Amount of Content sub-factor above caregivers—Content Diversity (7.7%, rank 5, vs. 5.6%, rank 6), Total Content Volume (2.7%, rank 11, vs. 1.6%, rank 18), and Content Duration (1.7%, rank 15, vs. 1.2%, rank 20)—whereas caregivers weighted every Quality of Content sub-factor above SLPs—Generalization (5.6%, rank 7, vs. 3.7%, rank 8), Facilitation of Communicative Interactions (2.5%, rank 12, vs. 1.3%, rank 18), and Induction of Engagement and Attention (2.2%, rank 14, vs. 1.0%, rank 20). All six comparisons ran in the direction indicated by the medium-level weights, showing that the two groups’ contrasting orientations toward content were expressed consistently throughout the hierarchy.

In sum, the two groups converged closely on which factors mattered most and agreed almost entirely on what mattered least, differing mainly in how strongly they weighted the child’s cognitive ability and device safety and, most systematically, in whether they weighted the availability of content or its transferability to everyday communication.

### 3.2. Priorities by Child Age Group Among Caregivers

#### 3.2.1. High-Level Categories

All three caregiver subgroups produced the same high-level ranking (Subject, Content, System), and the weights were of similar magnitude. Subject accounted for 66.1% of the total weight among caregivers of children in early childhood, 69.0% among caregivers of pre-school children, and 67.8% among caregivers of school-age children, followed in every subgroup by Content (17.7%, 19.3%, and 19.3%, respectively) and System (16.2%, 11.6%, and 12.9%, respectively). The child’s developmental stage therefore did not alter the overall structure of caregiver priorities. The only appreciable variation occurred in System, which caregivers of children in early childhood weighted 4.6 percentage points higher than caregivers of pre-school children—the largest between-subgroup difference at this level (Table 4).

#### 3.2.2. Medium-Level Categories

Rank order at the medium level was identical across the three age subgroups within Subject and within System, and differed only within Content. Within Subject, all subgroups placed Child first (62.8%, 64.2%, and 60.8% for caregivers of children in early childhood, pre-school, and school-age children, respectively), followed by SLP (19.3%, 19.1%, and 23.8%) and then Caregiver (17.9%, 16.7%, and 15.5%). Although the ordering was stable, weight shifted gradually with the child’s age: caregivers of school-age children assigned the most weight to the SLP and the least to themselves. A parallel pattern emerged within System, where all subgroups placed Device first (61.7%, 64.3%, and 54.5%), followed by Environment (20.8%, 21.2%, and 26.7%) and then Network Connectivity (17.5%, 14.5%, and 18.9%); caregivers of school-age children weighted Device markedly lower and Environment markedly higher than the two younger subgroups. Within Content, caregivers of children in early childhood and of school-age children favored Quality of Content (57.8% and 62.5%, respectively), whereas caregivers of pre-school children were nearly evenly divided and favored Amount of Content (50.8%; Table 5).

#### 3.2.3. Sub-Level Factors

The leading sub-level priorities were largely consistent across the three age subgroups; accordingly, only the factors that distinguish the subgroups are described here, with the complete rankings presented in Table 6. All three subgroups placed the child’s Age and Physical-Motor Development Level first by a wide margin (26.0%, 28.7%, and 26.5% for caregivers of children in early childhood, pre-school, and school-age children, respectively) and the child’s Device and Content Preferences second (7.8%, 8.7%, and 8.2%), and all three ranked the child’s Cognitive Ability and the SLP’s System Management Skills either third or fourth. The lowest-ranked factors were likewise common to the three subgroups: the Network Connectivity sub-factors and, within Environment, Private Space and Quiet Space each received no more than 1.6% of the total weight.

The remaining differences between the three age subgroups were ones of degree, and they formed a broadly consistent developmental gradient.

Caregiver-related sub-factors tended to decline in priority as the child’s age increased. The caregiver’s own Device Proficiency and Preferences held an almost identical rank across subgroups (5th, 6th, and 6th) but carried its lowest weight among caregivers of school-age children (4.9%, vs. 6.4% and 6.7%). The caregiver’s Role as an Assistant in the Metaverse ranked 9th and 10th among caregivers of children in early childhood and pre-school children and fell to 13th among caregivers of school-age children; its weight in the school-age subgroup, however, was identical to that in the early-childhood subgroup (3.1%) and higher than in the pre-school subgroup (2.6%), so this change reflects the rise of competing factors rather than a decline in its own importance. The caregiver’s Awareness of the Metaverse was similar throughout (13th, 2.3%; 12th, 2.2%; and 14th, 2.5%). Device Safety followed the same downward direction and did so monotonically, declining from 5.9% (rank 6) among caregivers of children in early childhood to 4.4% (rank 8) among caregivers of pre-school children and 3.4% (rank 10) among caregivers of school-age children.

Conversely, factors related to the SLP’s competencies and to the functional yield of intervention strengthened with the child’s age. The clearest instance was the SLP’s Ability to Use the Metaverse in Intervention, which entered the top ten only among caregivers of school-age children (4.7%, rank 7, vs. 2.5% and 2.2%, ranks 12 and 11)—the largest single shift observed. The SLP’s Data Awareness and Management Level carried its greatest weight in the same subgroup (3.9%, vs. 3.0% and 2.8%). Two Quality of Content sub-factors followed the same direction: Facilitation of Communicative Interactions (3.3%, vs. 2.5% and 2.2%) and Induction of Engagement and Attention (3.2%, vs. 2.1% and 1.9%). Generalization, although essentially stable in weight (5.7%, 5.4%, and 5.6%), rose from rank 7 to rank 5 in the school-age subgroup as competing factors declined.

One factor did not follow the age gradient. Content Diversity (under Amount of Content) peaked among caregivers of pre-school children (6.8%, rank 5) rather than varying monotonically, and was ranked 8th in both other subgroups (4.8% and 4.4%).

Tested against the individual weights, these subgroup differences were mostly small. The aggregate orderings of the 24 sub-factors corresponded closely across the three subgroups (Spearman ρ = 0.958, 0.957 and 0.965 for the three pairwise comparisons), which supports the description of the ranking as stable. Of the three high-level categories only System differed significantly across subgroups (Kruskal–Wallis, q = 0.001, ε^2^ = 0.069), none of the eight medium-level comparisons reached significance, and of the 24 sub-factors three did: the SLP’s System Management Skills (q = 0.033, ε^2^ = 0.047), Rapid and Appropriate Backup Systems (q = 0.042, ε^2^ = 0.042) and Content Diversity (q = 0.033, ε^2^ = 0.049). The effect sizes are small, and the remaining descriptive differences described above, including those in the caregiver’s assistant role and in Device Safety, were not statistically distinguishable and should be read as exploratory.

Because the caregiver sample included children with different diagnoses, priorities were also derived separately for the two subgroups large enough to support estimation. The aggregate orderings for children with developmental language disorder and children without a diagnosis corresponded closely (Spearman ρ = 0.961, *p* < 0.001) and the three highest-ranked sub-factors were identical in both, indicating that the diagnostic composition of the sample did not drive the reported priorities. The magnitudes of the individual weights did differ between these subgroups on 16 of the 24 sub-factors, with caregivers of children without a diagnosis assigning more weight to environmental and infrastructural factors such as Safe Space (rank-biserial r = −0.48) and Implementation of Secure Personal Data Protection Systems (r = −0.44). This difference cannot be attributed to diagnosis, however, because the two subgroups differ markedly in the age of the child (χ^2^ = 38.5, df = 2, *p* < 0.001): children without a diagnosis were concentrated in early childhood (72 of 122) whereas those with developmental language disorder were concentrated in the pre-school years (72 of 108). Diagnosis and developmental stage are therefore confounded in this sample and cannot be separated, which is stated in the Limitations.

## 4. Discussion

### 4.1. Principal Findings

This study examined and compared the factors that caregivers and SLPs regard as important when applying metaverse technology in children’s speech–language therapy. The two groups shared a strong child-centered orientation and agreed closely on both the most and the least important factors; within that shared structure, they differed in their expectations regarding intervention content and, to a lesser extent, the child’s cognitive ability and device safety. Among caregivers, priorities were stable in rank order across the child’s developmental stages, with graded differences in emphasis.

The clearest point of agreement was the primacy of the Subject domain and, within it, the Child sub-category. This finding suggests that the introduction of an immersive technology does not alter the client-centered foundation of speech–language therapy; rather, stakeholders expect metaverse-based services to be tailored to the characteristics and needs of individual children. This convergence further indicates that technology is viewed as a vehicle for, rather than a substitute for, developmentally grounded practice—an interpretation consistent with the family-centered orientation that characterizes pediatric rehabilitation more broadly [30].

### 4.2. Comparison with Prior Work

Child-related factors: Although both groups ranked the child’s Age and Physical-Motor Development Level first overall, they weighted the child domain differently: caregivers gave developmental-motor characteristics a larger share and ranked Cognitive Ability only fourth, whereas SLPs placed Cognitive Ability second. This difference may reflect their differing vantage points. Caregivers may emphasize physical and motor characteristics because these are readily observable in everyday contexts and are perceived as indicators of a child’s readiness to participate, whereas SLPs, who must select and adapt assessment and intervention approaches, may weight the cognitive abilities that support engagement with therapeutic tasks more heavily [31]. The implication is that child-related customization within metaverse platforms should accommodate both developmental-motor and cognitive characteristics.

Content-related factors: Within the Content domain, caregivers prioritized Quality of Content while SLPs prioritized Amount of Content, a divergence reproduced consistently at the sub-level: SLPs weighted all three Amount attributes more heavily—most notably Content Diversity—while caregivers weighted all three Quality attributes more heavily, most notably Generalization. Despite these opposing emphases, the two groups converged at both ends of the content ordering: Content Diversity and Generalization were the only content attributes to enter the top 10 in either group, and Content Duration remained low for both. Both groups thus valued content that applies across situations and supports meaningful communication over the sheer duration of exposure.

The two groups appear to have reached this shared frame for different reasons. For caregivers, the emphasis fell on what content achieves: their higher weighting of Generalization, Facilitation of Communicative Interactions, and Induction of Engagement and Attention suggests content is valued to the extent that it transfers to everyday communication and sustains participation, and the value they placed on diversity appears tied to the same desire for individualized intervention. This is consistent with Ju [32], who identified service quality as a key determinant of satisfaction among caregivers in developmental rehabilitation, and with international evidence that parents’ satisfaction is driven less by the volume of service than by whether care is individualized and responsive to family needs [30]. SLPs, by contrast, ranked content diversity highest of any content attribute in either group, but plausibly because planning and adapting interventions across a heterogeneous caseload requires a broad, flexible pool of resources. This reading aligns with Park et al. [33], who reported that insufficient assessment and intervention resources are a significant clinical challenge, and with survey evidence that limited time and caseload demands are among the most frequently reported barriers to evidence-based SLP practice [34]. Because participants were not asked to explain their judgments, these interpretations are inferences from the weighting pattern and warrant direct examination in future qualitative work.

These findings also speak to the observation that many existing metaverse- and VR-based interventions rely on restricted scenarios and repetitive procedures, limiting their ability to sustain engagement and promote problem-solving [35]. Such limitations run counter to what both groups considered important: if content diversity is a central shared priority, platforms should provide content spanning a wide range of contexts, developmental levels, and intervention goals rather than rehearsing a limited set of scenarios. Diverse, adaptable, and generalizable content should therefore be a key design priority.

System-related factors: The System findings reveal a consistent treatment of technology as a precondition rather than a primary benefit. System ranked lowest at the domain level for both groups, and most system sub-factors fell in the lower half of the ordering, including network speed and data protection. Within the domain, however, Device Safety stood clearly apart, entering the top 10 for both groups and outweighing every other system attribute by a wide margin; the marginally sharper safety concern among SLPs is consistent with their direct accountability for a child’s physical wellbeing during immersive use in clinical settings [16].

The pattern as a whole indicates that stakeholders do not regard technological sophistication as a therapeutic good in itself; they treat device safety, stable connectivity, and data privacy as background conditions that are assumed rather than actively demanded, but that must nonetheless be satisfied before clinical value can be realized [36,37,38,39]. This is not evidence that these conditions are unimportant. Connectivity quality has measurable consequences for children’s outcomes in technology-mediated learning [37], and survey evidence indicates that the public regards control over personal information as important even when it is not spontaneously raised [38,39]. The design consequence is that safety and privacy should be engineered as defaults rather than offered as configurable extras, since stakeholders appear unlikely to seek them out even though their absence would undermine the service.

Age-stratified caregiver priorities: The age-stratified analysis among caregivers revealed a broadly stable priority structure across developmental stages, with differences of degree rather than of kind. All three subgroups ranked the domains identically (Subject, Content, System), placed Child first within Subject and Device first within System, and shared the same leading sub-factors, indicating that the child-centered orientation of caregivers is common to caregivers of children at all three age bands.

Within this stable structure, several differences of degree were graded across the age-defined subgroups; because different caregivers were sampled in each subgroup, these are cross-sectional contrasts rather than developmental change. Device Safety was weighted progressively lower in the older subgroups, congruent with national injury surveillance data showing higher safety-accident rates among children aged 0–6 years than among those aged 7–14 years [40], with evidence that immersive VR use in young children carries small but non-negligible physical risks [16], and with the progressive accumulation of independent device exposure among older children reported in Korea [41] and internationally [42]. In the same direction, the caregiver’s Role as an Assistant in the Metaverse fell out of the top 10 only for caregivers of school-age children, mapping onto the caregiver’s shift from a hands-on co-participant [17,18,43] to a more distal supporter of an increasingly independent child; because the weight of this factor was essentially unchanged across subgroups, the rank change reflects the rise of competing factors rather than a decline in its own importance.

Conversely, factors related to therapeutic yield and clinical implementation rose with child age: Generalization rose in rank for school-age children, and both the SLP’s Ability to Use the Metaverse in Intervention and Data Awareness and Management Level carried their greatest weight in that group. This gradient is consistent with the changing demands of school-age intervention, in which targets increasingly concern transfer of skills to classroom and everyday communication and depend on the clinician’s ability to select, adapt, and monitor tools [31], and with evidence that caregivers’ evaluation of technology-mediated services is closely tied to the clinician’s competence in delivering them [43,44]. Content priorities shifted only modestly, with Content Duration remaining near the bottom for all three groups.

Taken together, these contrasts converge on a qualified takeaway: caregiver priorities appear anchored in a stable, child-centered core across developmental stages, while their emphasis migrates from protection and hands-on assistance toward therapeutic yield and independent, clinically supported use as the child develops. What this suggests is not separate platforms for each stage but a single, age-adaptive system that can tune its content, interaction demands, and safety scaffolding—from protective, closely supervised experiences for younger children toward more self-managed, generalization-oriented experiences for school-age children. Because these subgroup contrasts were tested inferentially and only System differed significantly at the high level (Kruskal–Wallis, q = 0.001, ε^2^ = 0.069), with three sub-factors reaching significance at small effect sizes and the remaining differences not statistically distinguishable, and because the school-age subgroup was the smallest (n = 41), this reading should be regarded as a hypothesis for prospective evaluation rather than an established developmental effect.

### 4.3. Clinical and Design Implications

These findings may also be read through technology-acceptance frameworks such as the Technology Acceptance Model (TAM) [45] and the Unified Theory of Acceptance and Use of Technology (UTAUT) [46], which hold that adoption is driven by perceived usefulness, usability, and contextual relevance. That participants weighted developmental characteristics, content quality, and clinical applicability above technological sophistication suggests that acceptance of metaverse-based speech–language therapy may depend more on perceived therapeutic value and developmental fit than on the novelty of the medium, although acceptance itself was not measured in this study. Within this frame, the safety and reliability factors function as the usability and facilitating-condition prerequisites that must be met before perceived usefulness can translate into sustained use—infrastructure that is invisible when it works and noticed only when it fails.

Read this way, the findings translate into three priorities that span clinical practice and platform design. First, developmentally staged content that tracks the migration of caregiver emphasis from protection toward generalization and independent use as the child matures. Second, dual-pathway content provision that offers SLP-facing quantity and diversity tools alongside caregiver-facing individualization features. Third, safety as a default that satisfies the consistently top-10 ranking of Device Safety across every stakeholder group without burdening users.

Importantly, the extensive convergence observed here—an identical domain ordering, nine shared factors among the top ten, and near-identical weighting of the least important factors—indicates that these are not competing requirements calling for separate products. The shared child-centered core indicates that a single, developmentally grounded service—rather than separate products for each group—may be able to address both stakeholders’ priorities, a design hypothesis consistent with the observed rankings rather than a finding established here; the divergences are localized to the content layer and to the relative emphasis on cognitive versus physical-motor characteristics, and are therefore addressable through configurable content pathways and role-specific interfaces within a single system.

### 4.4. Generalizability to Other Settings

This study was conducted in Korea, and the transferability of its findings differs across the priority structure. The dominant features—the primacy of the Subject domain, the concentration of weight on the child within it, the close convergence of caregiver and SLP priorities, and the treatment of the technology as a precondition rather than a benefit—are claims about how stakeholders reason about a therapy technology rather than about any particular feature of the Korean health system. They converge with family-centered principles established in Canadian pediatric rehabilitation [30] and with eHealth development frameworks formulated in Europe [19], both of which locate the determinants of uptake in the people a technology serves rather than in its technical properties. The age-related gradient likewise tracks the changing demands of intervention across development, and no evident mechanism would confine it to a single country.

Other parts of the structure are plainly contextual, and infrastructure is the clearest case. Network Speed and Stability received 1.3% and 1.2% of the total weight in both groups, and the three Network Connectivity sub-factors together accounted for only 2.2% in both groups. Korea has near-universal high-speed broadband [36], and the near-absence of connectivity from the priority structure is more plausibly read as evidence that the problem has already been solved than as evidence that it does not matter: where broadband access is uneven, it constrains technology-mediated learning directly [37]. In such settings connectivity would be expected to carry substantially more weight. The same reasoning applies to device cost, which is sensitive to how services are financed, and to caregivers’ device proficiency, which is elevated in a population with exceptionally high smartphone penetration [41]. Priorities elicited in one setting therefore disclose what has already been resolved there as much as what stakeholders value, and this asymmetry should be weighed whenever the present weights are transported elsewhere.

The hierarchy and the three-stage procedure that generated it are nevertheless reusable independently of the weights. Administering the same instrument in health systems with different financing arrangements, infrastructure, and caregiving norms would test directly which of the priorities reported here are structural features of stakeholder reasoning and which are artifacts of a high-connectivity, early-adopter setting. The weights reported here are offered as a first benchmark for that comparison.

These priorities must also be read against current guidance on screen exposure in early childhood. The World Health Organization recommends no more than one hour of sedentary screen time per day for children aged 2–4 years, with less being better, and no screen time for those under 2 [47]. A meta-analysis of screen use and child language reports that greater quantity of screen exposure is negatively associated with language outcomes, while the quality of the content and shared viewing with a caregiver are associated with better outcomes [48]. A more recent meta-analysis makes the same point about the context rather than the quantity of use, finding that educational content and co-use with an adult are associated with more favourable cognitive and psychosocial outcomes than solitary or background exposure [49]. Two implications follow for the priorities reported here. The weight caregivers and SLPs placed on Content Diversity, Generalization, and Induction of Engagement and Attention is consistent with that evidence, since these are properties of content quality rather than of exposure time. At the same time, the constraints bound what metaverse-based therapy can responsibly offer to the early-childhood group, whose priorities were elicited without reference to session duration; any implementation would need to specify session length and caregiver co-use explicitly, and to make that guidance available to caregivers and to the designers of such systems.

## 5. Limitations

Several limitations should be acknowledged.

Sampling and participants. Participants were recruited by convenience sampling concentrated in the Incheon, Gyeonggi, and Chungcheong regions, and the caregiver sample was predominantly mothers (81.2%), college graduates (84.0%), and aged 30–39 years (87.2%); the sample is therefore not representative, and the weights should not be read as population estimates. Because socioeconomic position shapes access to devices, home connectivity, and the time a caregiver can devote to supporting therapy, the priorities reported here may understate the weight that families with fewer resources would place on cost, equipment burden, and the demands made of the caregiver; sampling that reaches lower-socioeconomic and less-supported families is a specific requirement for further work. Eligibility was defined by language functioning rather than diagnosis, so the sample deliberately spans several etiologies (Table S1). This is consistent with the inclusion criterion and arguably strengthens ecological validity, since services are delivered to a clinically varied caseload, but it limits what can be said about diagnosis-specific priorities: severity was not recorded, and three of the five diagnostic categories (intellectual disability n = 14, autism spectrum disorder n = 5, speech sound disorder n = 1) were too small to analyze separately; the two largest, developmental language disorder (n = 108) and no diagnosis (n = 122), were compared. Whether priorities differ by diagnosis or severity therefore remains open. Diagnosis and developmental stage are also confounded: children without a formal diagnosis were concentrated in early childhood and those with developmental language disorder in the pre-school years, so the subgroup differences in weight magnitude reported in Section 3.2 cannot be attributed to diagnosis, and a design that crosses diagnosis with age band would be needed to separate them. The questionnaire recorded SLPs’ years of clinical experience but did not verify the grade of their national certificate, so the certification profile of that sample is unknown. Data were also collected in a single country, and the weights are conditioned on its infrastructure and service arrangements, as set out above.

Instrument and administration. The definition of one of the 24 sub-factors, Device Proficiency and Preferences, was omitted from the expert content-validity review, so its wording did not receive the scrutiny given to the other 23. Because the surveys were administered anonymously, no platform-level control against duplicate submission could be applied without linking responses to identifiable accounts. The one-voucher-per-number rule imposed by the grant’s disbursement procedure provided a partial check among respondents who supplied a contact number, but repeated participation could not be ruled out for those who did not, or for an individual using more than one number. The same constraint prevents any check of whether individuals took part in more than one stage.

Analysis. AHP yields relative priorities rather than parameter estimates with sampling distributions. The inferential comparisons reported here were therefore applied to individual-level weights that are constrained to sum to one within each cluster, so they indicate whether the groups differ in these samples rather than providing design-based inference to a wider population, and differences that did not reach significance should not be read as evidence of equivalence. The school-age subgroup was the smallest (n = 41), and the age-stratified contrasts should be treated with particular caution. Consistency was enforced at CR ≤0.20, a more permissive criterion than the conventional 0.10 [20,26]; the rationale is given in the Methods. Because the platform blocked submission until each comparison set met this criterion, all analyzed responses satisfied it by construction, and the small attrition from the submitted to the analytic sample was due to missing eligibility or classification items rather than inconsistency. One caveat follows: every comparison matrix here was of order two or three, for which Saaty recommends a stricter cut-off, so accepted responses may carry more internal inconsistency than in expert-panel studies. The platform did not log individual CR values, but because the judgments themselves were retained it was possible to recompute them post hoc and to test the sensitivity of the findings to the stricter threshold, as reported in the Methods.

Interpretation. Participants judged a described rather than an experienced technology. The priorities reported here are stated expectations elicited before use, and stated preferences do not always predict behavior once a system is in place. The Stage 3 survey did not record prior metaverse experience, so it cannot be determined whether responses differed between participants with and without previous exposure; given the established moderating influence of experience on technology acceptance [46], this is an important gap, and experience data were collected only at Stage 1, in a much smaller sample. Finally, the limited published literature on metaverse-based speech–language therapy constrained the scope of comparative interpretation, and several of the comparisons drawn above rely on adjacent studies (VR-based intervention, telepractice, and pediatric rehabilitation more generally).

Future research should examine how perceptions vary with metaverse experience, assess satisfaction among caregivers and SLPs who have actually used metaverse-based therapy, and test how perceived therapeutic value and developmental appropriateness shape adoption intentions, satisfaction, and sustained use across diverse age groups and clinical populations. Prospective studies with probability-based or stratified sampling, and with pre-registered subgroup comparisons, would allow the descriptive gradients reported here to be tested directly.

## 6. Conclusions

Caregivers and SLPs both place the child at the center of metaverse-based speech–language therapy, and their priority structures were closely aligned at every level of the hierarchy. Within this shared structure they diverged in emphasis: caregivers favored individualized, developmentally tailored content, whereas SLPs assigned relatively more weight to the child’s cognitive ability, content availability, and device safety. Among caregivers, emphasis differed in degree—though not in overall rank order—between the subgroups defined by the child’s age band, from early childhood to school age. These convergences and divergences suggest that a single, developmentally grounded service may be able to address both groups’ priorities—a design hypothesis consistent with the observed rankings rather than a finding established here—provided that it offers age-adaptive content pathways and role-differentiated content tools rather than assuming a uniform stakeholder model. Because the present findings are descriptive and derive from a non-probabilistic sample of participants who had not used such a platform, they are best treated as input for practice and design that should be tested. Their reach beyond Korea is uneven by design: the concentration of weight on the people involved in therapy is a claim about how stakeholders reason and should travel, whereas the negligible weight given to network connectivity may reflect infrastructure that is already well established in this setting—a reading that remains to be tested rather than one demonstrated *here*. Future research should evaluate how differentiated, developmentally responsive designs affect actual adoption, satisfaction, and therapeutic outcomes in clinical practice, and should administer the present hierarchy in health systems with different infrastructure and financing to establish which of these priorities are structural and which are contextual.

## Figures and Tables

**Table 1:** Priorities of high-level categories.

High-Level Category	Caregivers, Rank (Weight)	SLPs, Rank (Weight)
Subject	1 (0.678)	1 (0.674)
Content	2 (0.187)	2 (0.181)
System	3 (0.134)	3 (0.145)

**Table 2:** Priorities of medium-level categories. Weights may not sum to exactly 1 owing to rounding.

High-Level Category	Medium-Level Category	Caregivers, Rank (Weight)	SLPs, Rank (Weight)
Subject	Child	1 (0.631)	1 (0.643)
	Caregiver	3 (0.169)	3 (0.148)
	SLP	2 (0.199)	2 (0.209)
System	Device	1 (0.618)	1 (0.646)
	Network Connectivity	3 (0.163)	3 (0.154)
	Environment	2 (0.219)	2 (0.200)
Content	Amount of Content	2 (0.453)	1 (0.669)
	Quality of Content	1 (0.547)	2 (0.331)

**Table 3:** Priorities of sub-factors by caregivers and SLPs.

Medium-Level Category	Sub-Factor	Caregivers, Rank (Global; Local Weight)	SLPs, Rank (Global; Local Weight)
Child	Age and Physical-Motor Development Level	**1** (**0.274; 0.639**)	**1** (**0.248; 0.573**)
	Cognitive Ability	**4** (**0.071; 0.167**)	**2** (**0.093; 0.215**)
	Device and Content Preferences	**2** (**0.083; 0.194**)	**3** (**0.092; 0.212**)
Caregiver	Device Proficiency and Preferences	**5** (**0.063; 0.547**)	**6** (**0.061; 0.616**)
	Awareness of the Metaverse	13 (0.023; 0.200)	16 (0.016; 0.156)
	Role as an Assistant in the Metaverse	**10** (**0.029; 0.253**)	12 (0.023; 0.228)
SLP	System Management Skills	**3** (**0.078; 0.577**)	**4** (**0.082; 0.581**)
	Ability to Use the Metaverse in Intervention	11 (0.027; 0.197)	**10** (**0.029; 0.203**)
	Data Awareness and Management Level	**9** (**0.030; 0.225**)	**9** (**0.030; 0.216**)
Device	Device Safety	**8** (**0.048; 0.574**)	**7** (**0.060; 0.643**)
	Ease of Use	17 (0.017; 0.203)	17 (0.015; 0.162)
	Device Purchase and Maintenance Costs	15 (0.019; 0.223)	13 (0.018; 0.196)
Network Connectivity	Network Speed and Stability	19 (0.013; 0.582)	19 (0.012; 0.558)
	Rapid and Appropriate Backup Systems	24 (0.004; 0.190)	24 (0.004; 0.189)
	Implementation of Secure Personal Data Protection Systems	23 (0.005; 0.228)	22 (0.006; 0.253)
Environment	Safe Space	16 (0.018; 0.624)	14 (0.018; 0.610)
	Private Space	22 (0.005; 0.174)	23 (0.005; 0.158)
	Quiet Space	21 (0.006; 0.202)	21 (0.007; 0.232)
Amount of Content	Content Diversity	**6** (**0.056; 0.663**)	**5** (**0.077; 0.636**)
	Content Duration	20 (0.012; 0.146)	15 (0.017; 0.141)
	Total Content Volume	18 (0.016; 0.191)	11 (0.027; 0.223)
Quality of Content	Generalization	**7** (**0.056; 0.543**)	**8** (**0.037; 0.617**)
	Induction of Engagement and Attention	14 (0.022; 0.212)	20 (0.010; 0.159)
	Facilitation of Communicative Interactions	12 (0.025; 0.245)	18 (0.013; 0.225)

Values in bold indicate the 10 highest-ranked sub-factors within each group. Weights are global weights and sum to 1.00 within each group. Ranks were determined from unrounded weights; because weights are reported to three decimal places, factors with identical reported weights may hold different ranks, and tied ranks indicate weights that are equal at the precision used for ranking.

**Table 4:** Priorities of high-level categories by age group of children.

High-Level Category	Early Childhood, Rank (Weight)	Pre-School, Rank (Weight)	School-Age, Rank (Weight)
Subject	1 (0.661)	1 (0.690)	1 (0.678)
System	3 (0.162)	3 (0.116)	3 (0.129)
Content	2 (0.177)	2 (0.193)	2 (0.193)

**Table 5:** Priorities of medium-level categories by age group of children.

High-Level Category	Medium-Level Category	Early Childhood, Rank (Weight)	Pre-School, Rank (Weight)	School-Age, Rank (Weight)
Subject	Child	1 (0.628)	1 (0.642)	1 (0.608)
	Caregiver	3 (0.179)	3 (0.167)	3 (0.155)
	SLP	2 (0.193)	2 (0.191)	2 (0.238)
System	Device	1 (0.617)	1 (0.643)	1 (0.545)
	Network Connectivity	3 (0.175)	3 (0.145)	3 (0.189)
	Environment	2 (0.208)	2 (0.212)	2 (0.267)
Content	Amount of Content	2 (0.422)	1 (0.508)	2 (0.375)
	Quality of Content	1 (0.578)	2 (0.492)	1 (0.625)

**Table 6:** Priorities of sub-factors by age group of children.

Medium-Level Category	Sub-Factor	Early Childhood, Rank (Global; Local)	Pre-School, Rank (Global; Local)	School-Age, Rank (Global; Local)
Child	Age and Physical-Motor Development Level	**1** (**0.260; 0.627**)	**1** (**0.287; 0.648**)	**1** (**0.265; 0.644**)
	Cognitive Ability	**3** (**0.077; 0.185**)	**4** (**0.069; 0.155**)	**4** (**0.065; 0.158**)
	Device and Content Preferences	**2** (**0.078; 0.188**)	**2** (**0.087; 0.197**)	**2** (**0.082; 0.199**)
Caregiver	Device Proficiency and Preferences	**5** (**0.064; 0.545**)	**6** (**0.067; 0.579**)	**6** (**0.049; 0.465**)
	Awareness of the Metaverse	13 (0.023; 0.192)	12 (0.022; 0.193)	14 (0.025; 0.238)
	Role as an Assistant in the Metaverse	**9** (**0.031; 0.263**)	**10** (**0.026; 0.228**)	13 (0.031; 0.296)
SLP	System Management Skills	**4** (**0.073; 0.568**)	**3** (**0.082; 0.622**)	**3** (**0.075; 0.466**)
	Ability to Use the Metaverse in Intervention	12 (0.025; 0.197)	11 (0.022; 0.168)	**7** (**0.047; 0.295**)
	Data Awareness and Management Level	**10** (**0.030; 0.234**)	**9** (**0.028; 0.210**)	**9** (**0.039; 0.239**)
Device	Device Safety	**6** (**0.059; 0.595**)	**8** (**0.044; 0.584**)	**10** (**0.034; 0.492**)
	Ease of Use	17 (0.019; 0.190)	18 (0.015; 0.194)	16 (0.019; 0.265)
	Device Purchase and Maintenance Costs	15 (0.021; 0.215)	16 (0.017; 0.222)	17 (0.017; 0.242)
Network Connectivity	Network Speed and Stability	18 (0.016; 0.549)	20 (0.010; 0.606)	19 (0.014; 0.590)
	Rapid and Appropriate Backup Systems	22 (0.006; 0.217)	24 (0.003; 0.170)	24 (0.004; 0.185)
	Implementation of Secure Personal Data Protection Systems	21 (0.007; 0.233)	23 (0.004; 0.224)	22 (0.005; 0.226)
Environment	Safe Space	14 (0.022; 0.639)	17 (0.015; 0.609)	15 (0.022; 0.628)
	Private Space	23 (0.006; 0.181)	22 (0.004; 0.176)	23 (0.005; 0.151)
	Quiet Space	24 (0.006; 0.180)	21 (0.005; 0.215)	21 (0.008; 0.222)
Amount of Content	Content Diversity	**8** (**0.048; 0.647**)	**5** (**0.068; 0.693**)	**8** (**0.044; 0.609**)
	Content Duration	20 (0.013; 0.170)	19 (0.012; 0.122)	20 (0.012; 0.167)
	Total Content Volume	19 (0.014; 0.182)	15 (0.018; 0.186)	18 (0.016; 0.224)
Quality of Content	Generalization	**7** (**0.057; 0.552**)	**7** (**0.054; 0.565**)	**5** (**0.056; 0.462**)
	Induction of Engagement and Attention	16 (0.021; 0.202)	14 (0.019; 0.203)	12 (0.032; 0.262)
	Facilitation of Communicative Interactions	11 (0.025; 0.246)	13 (0.022; 0.232)	11 (0.033; 0.276)

Values in bold indicate the 10 highest-ranked sub-factors within each subgroup. Weights are global weights and sum to 1.00 within each subgroup. Ranks were determined from unrounded weights; because weights are reported to three decimal places, factors with identical reported weights may hold different ranks, and tied ranks indicate weights that are equal at the precision used for ranking.

## Data Availability

The individual-level pairwise-comparison data presented in this study, together with the analysis script that reproduces all reported weights, confidence intervals and tests, are available on request from the corresponding author. The individual-level data are not publicly available due to privacy restrictions and participant consent; the script is not subject to those restrictions and can be deposited in a public repository if required.

## Supplementary Materials

The following supporting information can be downloaded at: https://www.mdpi.com/article/10.3390/children13101348/s1: Table S1: Characteristics of participants (caregivers, N = 250; speech–language pathologists [SLPs], N = 67); Stage 3 explanatory material and questionnaire (Korean original with English translation); Table S2: Aggregated pairwise-comparison matrices, with eigenvalues, consistency indices and derived weights; Table S3: Individual-level comparisons of priority weights; Table S4: Expert content validity ratings of the sub-factor definitions; Table S5: Mapping from Stage 1 concepts through Stage 2 options to the Stage 3 sub-factors; Table S6: Bootstrap 95% confidence intervals for the aggregate AHP weights.
