# Supplementary material for: Caregiver and Clinician Priorities for User-Centered Metaverse-Based Speech–Language Therapy: An Analytic Hierarchy Process Study
Source: Children (Basel). 2026 Oct 4;13(10):1348. doi: 10.3390/children13101348 (PMC13703426; doi:10.3390/children13101348)
Supplement: Supplementary file 1 [file children-13-01348-s001.zip › children-4536795-supplementary.pdf]

Supplementary Materials

Caregiver and Clinician Priorities for User-Centered Metaverse-Based Speech-Language Therapy: An Analytic Hierarchy Process Study

Tables S1 to S6 follow.

Table S1. Characteristics of participants (caregivers, N = 250; speech-language pathologists [SLPs], N = 67).

Group	Characteristic	Category	*n* (%)
Caregivers (*N*=250)	Relationship with child	Father	46 (18.4)
		Mother	203 (81.2)
		Missing	1 (0.4)
	Age range (years)	25–29	18 (7.2)
		30–34	132 (52.8)
		35–39	86 (34.4)
		40–44	14 (5.6)
	Education	Less than high school diploma	1 (0.4)
		High school diploma	32 (12.8)
		Enrolled in college	5 (2.0)
		College graduate	210 (84.0)
		Graduate degree or higher	2 (0.8)
	Gender of child	Male	102 (40.8)
		Female	147 (58.8)
		Missing	1 (0.4)
	Age of child	Early childhood (0–3 years)	95 (38.0)
		Pre-school (4–6 years)	114 (45.6)
		School-age (7–12 years)	41 (16.4)
	Child's disability diagnosis	No disability diagnosis	122 (48.8)
		Developmental language disorder	108 (43.2)
		Intellectual disability	14 (5.6)
		Autism spectrum disorder	5 (2.0)
		Speech sound disorder	1 (0.4)
SLPs (*N*=67)	Gender	Male	25 (37.3)
		Female	42 (62.7)
	Education	College graduate	26 (38.8)
		Graduate degree or higher	41 (61.2)
	Clinical experience (years)	Less than 1	2 (3.0)
		1–3	11 (16.4)
		4–6	35 (52.2)
		7–9	12 (17.9)
		10 or more	7 (10.4)

Data missing for two different caregivers: one did not report their relationship to the child, and another did not report the child's gender. Because neither variable was used in the AHP computations or in the age-based stratification, retaining these two records had no effect on the analyses. All children had a language development deficit and were receiving speech-language therapy at the time of the survey; "no disability diagnosis" denotes children who had not

received a formal disability diagnosis. Diagnoses were reported by caregivers and were not clinically verified; the item asked for a single primary diagnosis, so the categories are mutually exclusive and comorbid conditions were not captured.

Table S2. Aggregated pairwise-comparison matrices, with eigenvalues, consistency indices and derived weights.

Each matrix is the element-wise geometric mean of all respondents' judgments in that group (aggregation of individual judgments). Weights are the normalized principal eigenvector. Matrices of order two are consistent by construction (CR = 0).

Caregivers (*n* = 250)

Cluster	Element	(1)	(2)	(3)	Weight	λmax	CI	CR
Goal	Subject	1.000	4.789	3.817	0.6783	3.003	0.0014	0.0024
	System	0.209	1.000	0.681	0.1344			
	Content	0.262	1.468	1.000	0.1873			
Subject	Child	1.000	3.688	3.202	0.6314	3.000	0.0001	0.0001
	Caregiver	0.271	1.000	0.842	0.1694			
	SLP	0.312	1.188	1.000	0.1992			
System	Device	1.000	3.547	3.005	0.6176	3.004	0.0020	0.0035
	Network Connectivity	0.282	1.000	0.700	0.1634			
	Environment	0.333	1.429	1.000	0.2190			
Content	Amount of Content	1.000	0.828		0.4530	—	—	0
	Quality of Content	1.208	1.000		0.5470			
Child	Age and Physical-Motor Development Level	1.000	3.683	3.433	0.6394	3.002	0.0008	0.0014
	Cognitive Ability	0.272	1.000	0.825	0.1666			
	Device and Content Preferences	0.291	1.213	1.000	0.1940			
Caregiver	Device Proficiency and Preferences	1.000	2.604	2.279	0.5475	3.002	0.0012	0.0021
	Awareness of the Metaverse	0.384	1.000	0.753	0.2000			
	Role as an Assistant in the Metaverse	0.439	1.327	1.000	0.2525			
SLP	System Management Skills	1.000	2.804	2.672	0.5773	3.002	0.0009	0.0015
	Ability to Use the Metaverse in Intervention	0.357	1.000	0.841	0.1974			
	Data Awareness and Management Level	0.374	1.190	1.000	0.2253			
Device	Device Safety	1.000	2.725	2.662	0.5736	3.001	0.0006	0.0011
	Ease of Use	0.367	1.000	0.878	0.2031			
	Device Purchase and Maintenance Costs	0.376	1.139	1.000	0.2233			
Network Connectivity	Network Speed and Stability	1.000	2.951	2.655	0.5822	3.002	0.0008	0.0014
	Rapid and Appropriate Backup Systems	0.339	1.000	0.799	0.1896			
	Implementation of Secure Personal Data Protection Systems	0.377	1.252	1.000	0.2282			
Environment	Safe Space	1.000	3.504	3.168	0.6242	3.001	0.0003	0.0006
	Private Space	0.285	1.000	0.838	0.1737			
	Quiet Space	0.316	1.194	1.000	0.2021			
Amount of Content	Content Diversity	1.000	4.365	3.616	0.6632	3.002	0.0008	0.0013
	Content Duration	0.229	1.000	0.736	0.1460			
	Total Content Volume	0.277	1.359	1.000	0.1908			
Quality of Content	Generalization	1.000	2.507	2.273	0.5434	3.001	0.0003	0.0005

Cluster	Element	(1)	(2)	(3)	Weight	λmax	CI	CR
	Induction of Engagement and Attention	0.399	1.000	0.845	0.2117			
	Facilitation of Communicative Interactions	0.440	1.184	1.000	0.2448			

SLPs (*n* = 67)

Cluster	Element	(1)	(2)	(3)	Weight	λmax	CI	CR
Goal	Subject	1.000	4.411	3.920	0.6739	3.003	0.0014	0.0024
	System	0.227	1.000	0.758	0.1449			
	Content	0.255	1.318	1.000	0.1812			
Subject	Child	1.000	3.980	3.363	0.6432	3.008	0.0039	0.0067
	Caregiver	0.251	1.000	0.648	0.1479			
	SLP	0.297	1.542	1.000	0.2089			
System	Device	1.000	3.932	3.454	0.6462	3.005	0.0022	0.0038
	Network Connectivity	0.254	1.000	0.719	0.1537			
	Environment	0.290	1.391	1.000	0.2000			
Content	Amount of Content	1.000	2.026		0.6695	—	—	0
	Quality of Content	0.494	1.000		0.3305			
Child	Age and Physical-Motor Development Level	1.000	2.445	2.947	0.5730	3.008	0.0038	0.0066
	Cognitive Ability	0.409	1.000	0.926	0.2147			
	Device and Content Preferences	0.339	1.079	1.000	0.2123			
Caregiver	Device Proficiency and Preferences	1.000	3.580	2.981	0.6160	3.010	0.0049	0.0084
	Awareness of the Metaverse	0.279	1.000	0.620	0.1559			
	Role as an Assistant in the Metaverse	0.336	1.614	1.000	0.2281			
SLP	System Management Skills	1.000	2.715	2.845	0.5814	3.003	0.0015	0.0026
	Ability to Use the Metaverse in Intervention	0.368	1.000	0.889	0.2027			
	Data Awareness and Management Level	0.351	1.125	1.000	0.2159			
Device	Device Safety	1.000	3.594	3.641	0.6429	3.010	0.0052	0.0090
	Ease of Use	0.278	1.000	0.746	0.1615			
	Device Purchase and Maintenance Costs	0.275	1.341	1.000	0.1956			
Network Connectivity	Network Speed and Stability	1.000	2.861	2.283	0.5584	3.001	0.0005	0.0009
	Rapid and Appropriate Backup Systems	0.350	1.000	0.725	0.1890			
	Implementation of Secure Personal Data Protection Systems	0.438	1.379	1.000	0.2526			
Environment	Safe Space	1.000	3.481	2.906	0.6096	3.010	0.0051	0.0087
	Private Space	0.287	1.000	0.618	0.1584			
	Quiet Space	0.344	1.619	1.000	0.2320			
Amount of Content	Content Diversity	1.000	4.045	3.179	0.6359	3.012	0.0060	0.0103
	Content Duration	0.247	1.000	0.566	0.1409			
	Total Content Volume	0.315	1.767	1.000	0.2232			
Quality of Content	Generalization	1.000	3.701	2.882	0.6167	3.002	0.0012	0.0020
	Induction of Engagement and Attention	0.270	1.000	0.674	0.1588			
	Facilitation of Communicative Interactions	0.347	1.484	1.000	0.2246			

Table S3. Individual-level comparisons of priority weights.

Caregivers vs SLPs: Mann–Whitney U tests on individual weights; median (IQR) shown; 95% CI is the bootstrap interval for the difference in means (2,000 resamples); r is the rank-biserial correlation. Age subgroups: Kruskal–Wallis tests across early childhood (n = 95), pre-school (n = 114) and school-age (n = 41); ε^2 is epsilon squared. Diagnosis: developmental language disorder (n = 108) vs no diagnosis (n = 122). q values are Benjamini–Hochberg adjusted within each level.

High-level categories

Item	Caregivers median (IQR)	SLPs median (IQR)	Diff. 95% CI	q	r	q (age)	ε^2	q (dx)	r (dx)
Subject	0.710 (0.639–0.789)	0.732 (0.679–0.800)	-0.044, +0.075	0.522	-0.05	0.878	0.001	**0.000**	+0.32
System	0.100 (0.091–0.142)	0.091 (0.088–0.117)	-0.077, +0.022	0.522	+0.06	**0.001**	0.069	**0.000**	-0.53
Content	0.170 (0.101–0.230)	0.135 (0.110–0.200)	-0.029, +0.045	0.434	+0.12	0.349	0.012	0.178	-0.10

Medium-level categories (local weights)

Item	Caregivers median (IQR)	SLPs median (IQR)	Diff. 95% CI	q	r	q (age)	ε^2	q (dx)	r (dx)
Child	0.701 (0.550–0.800)	0.726 (0.615–0.800)	-0.069, +0.059	0.673	-0.04	0.598	0.004	**0.000**	+0.32
Caregiver	0.103 (0.091–0.174)	0.100 (0.091–0.121)	-0.029, +0.064	0.093	+0.17	0.440	0.010	**0.000**	-0.37
SLP	0.156 (0.097–0.260)	0.156 (0.100–0.233)	-0.055, +0.026	0.758	-0.03	0.440	0.008	**0.005**	-0.23
Device	0.691 (0.510–0.778)	0.710 (0.583–0.784)	-0.077, +0.046	0.541	-0.07	0.440	0.008	**0.000**	+0.30
Network Connectivity	0.108 (0.091–0.178)	0.100 (0.091–0.130)	-0.047, +0.049	0.353	+0.10	0.265	0.027	**0.000**	-0.39
Environment	0.178 (0.108–0.258)	0.143 (0.101–0.219)	-0.033, +0.056	0.195	+0.13	0.440	0.009	**0.038**	-0.16
Amount of Content	0.200 (0.167–0.875)	0.833 (0.250–0.875)	-0.271, -0.097	**0.039**	-0.20	0.440	0.014	**0.020**	+0.18
Quality of Content	0.800 (0.125–0.833)	0.167 (0.125–0.750)	+0.097, +0.271	**0.039**	+0.20	0.440	0.014	**0.020**	-0.18

Sub-factors (global weights)

Item	Caregivers median (IQR)	SLPs median (IQR)	Diff. 95% CI	q	r	q (age)	ε^2	q (dx)	r (dx)
Age and Physical-Motor Development Level	0.311 (0.062–0.489)	0.317 (0.045–0.411)	-0.027, +0.078	0.620	+0.08	0.927	0.001	**0.001**	+0.27
Cognitive Ability	0.061 (0.036–0.067)	0.056 (0.038–0.068)	-0.046, +0.004	0.910	-0.01	0.998	0.000	0.805	-0.02
Device and Content Preferences	0.066 (0.044–0.093)	0.071 (0.050–0.102)	-0.040, -0.000	0.745	-0.06	0.085	0.031	0.135	+0.13
Device Proficiency and Preferences	0.047 (0.032–0.061)	0.050 (0.030–0.058)	-0.020, +0.009	0.806	+0.03	0.628	0.008	0.805	-0.02
Awareness of the Metaverse	0.009 (0.007–0.026)	0.007 (0.006–0.015)	+0.011, +0.028	0.063	+0.19	0.628	0.007	**0.000**	-0.34
Role as an Assistant in the Metaverse	0.015 (0.009–0.029)	0.013 (0.008–0.022)	+0.006, +0.017	0.345	+0.13	0.628	0.008	**0.000**	-0.33
System Management Skills	0.061 (0.035–0.090)	0.066 (0.019–0.097)	-0.022, +0.009	0.806	-0.04	**0.033**	0.047	0.299	+0.08
Ability to Use the Metaverse in Intervention	0.014 (0.008–0.022)	0.014 (0.008–0.028)	-0.008, +0.013	0.916	-0.01	0.056	0.038	**0.042**	-0.17
Data Awareness and Management Level	0.018 (0.008–0.039)	0.020 (0.009–0.034)	-0.004, +0.012	0.806	+0.04	0.785	0.003	**0.023**	-0.19
Device Safety	0.044 (0.020–0.061)	0.048 (0.017–0.063)	-0.043, -0.004	0.414	-0.11	0.258	0.018	0.173	-0.11
Ease of Use	0.008 (0.006–0.018)	0.007 (0.005–0.014)	-0.006, +0.010	0.414	+0.10	0.195	0.021	**0.001**	-0.28

Item	Caregivers median (IQR)	SLPs median (IQR)	Diff. 95% CI	q	r	q (age)	ε²	q (dx)	r (dx)
Device Purchase and Maintenance Costs	0.012 (0.007–0.018)	0.011 (0.007–0.016)	-0.006, +0.006	0.801	+0.05	0.307	0.015	**0.000**	-0.38
Network Speed and Stability	0.007 (0.005–0.015)	0.007 (0.004–0.010)	-0.003, +0.006	0.414	+0.10	0.183	0.022	**0.000**	-0.33
Rapid and Appropriate Backup Systems	0.002 (0.001–0.006)	0.001 (0.001–0.005)	-0.002, +0.008	0.806	+0.04	**0.042**	0.042	**0.000**	-0.41
Implementation of Secure Personal Data Protection Systems	0.003 (0.001–0.006)	0.002 (0.001–0.005)	-0.026, +0.006	0.910	+0.02	0.183	0.023	**0.000**	-0.44
Safe Space	0.011 (0.007–0.019)	0.010 (0.007–0.015)	-0.022, +0.005	0.414	+0.10	0.085	0.031	**0.000**	-0.48
Private Space	0.003 (0.001–0.009)	0.003 (0.001–0.005)	+0.001, +0.006	0.637	+0.07	0.628	0.007	**0.000**	-0.36
Quiet Space	0.004 (0.001–0.008)	0.004 (0.001–0.007)	-0.005, +0.003	0.910	+0.02	0.662	0.005	**0.000**	-0.29
Content Diversity	0.032 (0.021–0.067)	0.066 (0.015–0.090)	-0.028, -0.007	**0.035**	-0.21	**0.033**	0.049	**0.016**	+0.20
Content Duration	0.007 (0.004–0.012)	0.010 (0.007–0.017)	-0.006, +0.001	**0.012**	-0.25	0.785	0.003	0.173	-0.11
Total Content Volume	0.009 (0.006–0.013)	0.013 (0.009–0.036)	-0.013, -0.002	**0.008**	-0.28	0.258	0.017	0.173	+0.11
Generalization	0.025 (0.011–0.100)	0.018 (0.010–0.048)	-0.015, +0.024	0.330	+0.13	0.927	0.001	0.134	-0.13
Induction of Engagement and Attention	0.014 (0.002–0.046)	0.004 (0.002–0.009)	+0.007, +0.034	**0.008**	+0.27	0.645	0.006	**0.007**	-0.22
Facilitation of Communicative Interactions	0.022 (0.003–0.050)	0.006 (0.002–0.017)	-0.004, +0.021	**0.033**	+0.22	0.628	0.007	**0.042**	-0.17

Bold q values are significant at $q < 0.05$.

Table S4. Expert content validity ratings of the sub-factor definitions.

Four speech-language pathologists rated each definition on a 5-point scale. I-CVI is the proportion of raters awarding 4 or 5. Retention required an item mean of 4.00 or above and an I-CVI of 0.75 or above; every rated item met both criteria. The definition of Device Proficiency and Preferences was inadvertently omitted from the review and is therefore not listed.

#	Sub-factor definition	R1	R2	R3	R4	Mean	I-CVI
1	Age and Physical-Motor Development Level	5	4	5	4	4.50	1.00
2	Cognitive Ability	5	5	5	4	4.75	1.00
3	Device and Content Preferences	5	5	5	3	4.50	0.75
4	Awareness of the Metaverse	5	4	5	4	4.50	1.00
5	Role as an Assistant in the Metaverse	5	5	5	4	4.75	1.00
6	System Management Skills	5	5	5	5	5.00	1.00
7	Ability to Use the Metaverse in Intervention	5	3	5	4	4.25	0.75
8	Data Awareness and Management Level	5	5	5	3	4.50	0.75
9	Device Safety	5	5	5	4	4.75	1.00
10	Ease of Use	5	5	5	3	4.50	0.75
11	Device Purchase and Maintenance Costs	5	5	5	3	4.50	0.75
12	Network Speed and Stability	5	5	5	2	4.25	0.75
13	Rapid and Appropriate Backup Systems	5	5	5	2	4.25	0.75
14	Implementation of Secure Personal Data Protection Systems	5	5	5	2	4.25	0.75
15	Safe Space	5	5	5	4	4.75	1.00
16	Private Space	5	5	5	3	4.50	0.75
17	Quiet Space	5	5	5	4	4.75	1.00
18	Content Diversity	5	5	5	4	4.75	1.00
19	Content Duration	5	5	5	5	5.00	1.00
20	Total Content Volume	5	5	5	3	4.50	0.75
21	Generalization	5	5	5	5	5.00	1.00
22	Induction of Engagement and Attention	5	5	5	4	4.75	1.00
23	Facilitation of Communicative Interactions	5	5	5	4	4.75	1.00
	**Overall**					**4.61**	**S-CVI/Ave 0.891**

Table S5. Mapping from Stage 1 concepts through Stage 2 options to the Stage 3 sub-factors.

Stage 1 concepts were derived by independent extraction from the open-ended responses of 17 caregivers and 6 SLPs, then merged into a single coding sheet. Stage 2 selection rates are based on the combined sample of 107 respondents (48 caregivers, 59 SLPs). Representative examples are shown; the last row illustrates an item retained under the triangulation rule despite a low selection rate.

Stage 1 concept (coding category)	Stage 2 option	Selection rate	Stage 3 sub-factor (parent category)
Child developmental level (cognitive, language, fine-motor, sensory, social)	The child's cognitive / language / fine-motor / sensory / social developmental level	69.2%	Age and Physical-Motor Development Level; Cognitive Ability (Child) — separated into physical-motor and cognitive aspects when the hierarchy was built
Engagement; content-related (elements that hold attention)	Engagement induced through diverse content	57.0%	Induction of Engagement and Attention (Quality of Content); Device and Content Preferences (Child)
Generalization (application to everyday life)	Ease of application to everyday life (generalization)	52.3%	Generalization (Quality of Content)
Interaction and rapport; emotion	Interaction and sharing of emotion (rapport building)	56.1%	Facilitation of Communicative Interactions (Quality of Content)
Caregiver competence (devices)	The caregiver's device skills and ability to use the metaverse	49.5%	Device Proficiency and Preferences (Caregiver)
Caregiver awareness	Positive awareness of the metaverse	30.8%	Awareness of the Metaverse (Caregiver)
Use of data (collection, analysis, evaluation)	Data can be analyzed and used	62.6%	Data Awareness and Management Level (SLP)
Therapist competence (devices, platform); device management	Strengthening the therapist's competence with devices and platforms	27.1%	System Management Skills (SLP)
Ease of use (simple operation, accessibility)	Convenience of device operation	65.4%	Ease of Use (Device)
Cost (device purchase and upkeep)	Financial capacity for device purchase and maintenance	39.3%	Device Purchase and Maintenance Costs (Device)
Space and time (a large, safe place)	A large, safe space where the device can be set up and used	43.9%	Safe Space (Environment)
Platform stability; internet connection	An intuitive and stable platform	60.7%	Network Speed and Stability (Network Connectivity)
Concerns (protection of personal data)	(low frequency — retained under the triangulation rule)	—	Implementation of Secure Personal Data Protection Systems (Network Connectivity)

Table S6. Bootstrap 95% confidence intervals for the aggregate AHP weights.

Estimand: the aggregate weight obtained by aggregating individual pairwise judgments with the geometric mean (aggregation of individual judgments, AIJ) and deriving the normalized principal eigenvector — that is, the quantity reported in Tables 1–3. Resampling: respondents were resampled with replacement within each group (2,000 resamples; caregivers n = 250, SLPs n = 67) and the full AIJ pipeline was re-run on every resample, so the intervals propagate uncertainty through aggregation, eigenvector extraction and synthesis. Intervals are percentile intervals. These intervals are distinct from those in Table S3, which are for the difference in means of individual-level weights between the two groups and are obtained by resampling the individual weights themselves. High-level and medium-level entries are local weights within their parent cluster; sub-factor entries are global weights. The analysis script that produced this table is available from the corresponding author on reasonable request.

Caregivers (*n* = 250)

Item	Weight	95% CI
High-level category		
Subject	0.678	[0.653, 0.699]
System	0.134	[0.124, 0.147]
Content	0.187	[0.173, 0.202]
Medium-level category (local weight)		
Child	0.631	[0.595, 0.663]
Caregiver	0.169	[0.153, 0.189]
SLP	0.199	[0.181, 0.219]
Device	0.618	[0.583, 0.649]
Network Connectivity	0.163	[0.148, 0.181]
Environment	0.219	[0.200, 0.240]
Amount of Content	0.453	[0.401, 0.506]
Quality of Content	0.547	[0.494, 0.599]
Sub-factor (global weight)		
Age and Physical-Motor Development Level	0.274	[0.246, 0.301]
Cognitive Ability	0.071	[0.064, 0.079]
Device and Content Preferences	0.083	[0.076, 0.089]
Device Proficiency and Preferences	0.063	[0.057, 0.069]
Awareness of the Metaverse	0.023	[0.019, 0.028]
Role as an Assistant in the Metaverse	0.029	[0.025, 0.034]
System Management Skills	0.078	[0.070, 0.087]
Ability to Use the Metaverse in Intervention	0.027	[0.023, 0.031]
Data Awareness and Management Level	0.030	[0.026, 0.035]
Device Safety	0.048	[0.043, 0.053]
Ease of Use	0.017	[0.015, 0.020]
Device Purchase and Maintenance Costs	0.019	[0.016, 0.021]
Network Speed and Stability	0.013	[0.011, 0.015]
Rapid and Appropriate Backup Systems	0.004	[0.003, 0.005]
Implementation of Secure Personal Data Protection Systems	0.005	[0.004, 0.006]
Safe Space	0.018	[0.016, 0.021]
Private Space	0.005	[0.004, 0.006]
Quiet Space	0.006	[0.005, 0.007]
Content Diversity	0.056	[0.050, 0.062]
Content Duration	0.012	[0.011, 0.014]

Total Content Volume	0.016	[0.014, 0.018]
Generalization	0.056	[0.049, 0.063]
Induction of Engagement and Attention	0.022	[0.017, 0.027]
Facilitation of Communicative Interactions	0.025	[0.020, 0.031]

Speech-language pathologists (*n* = 67)

**Item**	**Weight**	**95% CI**
High-level category		
Subject	0.674	[0.614, 0.721]
System	0.145	[0.116, 0.184]
Content	0.181	[0.155, 0.212]
Medium-level category (local weight)		
Child	0.643	[0.577, 0.697]
Caregiver	0.148	[0.119, 0.185]
SLP	0.209	[0.174, 0.249]
Device	0.646	[0.587, 0.696]
Network Connectivity	0.154	[0.125, 0.191]
Environment	0.200	[0.169, 0.241]
Amount of Content	0.669	[0.574, 0.750]
Quality of Content	0.331	[0.250, 0.426]
Sub-factor (global weight)		
Age and Physical-Motor Development Level	0.248	[0.193, 0.302]
Cognitive Ability	0.093	[0.073, 0.117]
Device and Content Preferences	0.092	[0.072, 0.113]
Device Proficiency and Preferences	0.061	[0.049, 0.076]
Awareness of the Metaverse	0.016	[0.012, 0.021]
Role as an Assistant in the Metaverse	0.023	[0.018, 0.029]
System Management Skills	0.082	[0.065, 0.100]
Ability to Use the Metaverse in Intervention	0.029	[0.021, 0.038]
Data Awareness and Management Level	0.030	[0.024, 0.038]
Device Safety	0.060	[0.047, 0.078]
Ease of Use	0.015	[0.011, 0.020]
Device Purchase and Maintenance Costs	0.018	[0.014, 0.024]
Network Speed and Stability	0.012	[0.009, 0.017]
Rapid and Appropriate Backup Systems	0.004	[0.003, 0.007]
Implementation of Secure Personal Data Protection Systems	0.006	[0.004, 0.009]
Safe Space	0.018	[0.013, 0.025]
Private Space	0.005	[0.003, 0.006]
Quiet Space	0.007	[0.005, 0.010]
Content Diversity	0.077	[0.064, 0.090]
Content Duration	0.017	[0.013, 0.022]
Total Content Volume	0.027	[0.021, 0.034]
Generalization	0.037	[0.025, 0.054]
Induction of Engagement and Attention	0.010	[0.006, 0.014]
Facilitation of Communicative Interactions	0.013	[0.009, 0.021]
