# Supplementary material for: Caregiver and Clinician Priorities for User-Centered Metaverse-Based Speech–Language Therapy: An Analytic Hierarchy Process Study
Source: Children (Basel). 2026 Oct 4;13(10):1348. doi: 10.3390/children13101348 (PMC13703426; doi:10.3390/children13101348)
Supplement: Supplementary file 1 [file children-13-01348-s001.zip › Supplementary_Questionnaire_corrected.pdf]

Supplementary Materials

Explanatory Material and Stage 3 Questionnaire

Manuscript ID: children-4536795

This file reproduces, without alteration, the explanatory material shown to participants and the Stage 3 questionnaire used to elicit the pairwise comparisons. The Korean originals appear first; an English translation of the instructions and of the operational definitions follows at the end.

Contents

1. Participant information sheet and explanatory material on the metaverse (Korean originals, as presented to participants; two sheets)
2. Stage 3 questionnaire, caregiver version (Korean original, 8 pages)
3. Stage 3 questionnaire, SLP version — demographic items only
4. English translation of the instructions and operational definitions

Notes

Explanatory material. Two sheets were presented to participants: the first sets out the purpose of the study, the sequence of survey rounds, the eligibility criteria, the closing date, and the QR codes for the caregiver and SLP versions; the second explains what the metaverse is and describes its four types. The same standardized material was presented at Stages 1 and 2, and the Stage 3 questionnaire opened with an equivalent introduction, so participants at every stage judged the same described system. The material was prepared by the Department of Communication Disorders, The Graduate School, Korea Nazarene University, under the supervision of Professor Gyung Hun Hong, with Ha-Rim Kim, Jeong-Eun Kim and Jin-Soo Lee; the contact line at the foot of each sheet has been annotated accordingly.

Consistency criterion. The printed instruction sheet reproduced here states the conventional criterion of 0.10, which is the wording commonly used in AHP questionnaires. The threshold actually implemented in the SSRA platform, and applied in real time to every comparison set, was CR ≤ 0.20, as reported in Section 2.6 of the manuscript. Respondents were not asked to compute or interpret the index themselves; the platform evaluated it and prompted revision when the criterion was not met. The distribution of CR values recomputed from the retained judgments confirms that 0.20 was the criterion in force: 83 caregivers (33.2%) and 35 SLPs (52.2%) submitted responses whose least consistent matrix exceeded 0.10.

Two versions of the questionnaire. The caregiver and SLP versions used the same 12 clusters, the same 24 sub-factors and operational definitions, the same item order, and the same scale anchors. They differed only in the demographic items, and only the differing items of the SLP version are reproduced here. Items 1 to 4 (gender, age band, education, region) and the final item requesting a telephone number for the voucher were identical in both versions.

1. Participant information sheet and explanatory material

Korean originals, as presented to participants. An English translation of the content appears in Section 4.

메타버스를 활용한 언어재활에 대한 양육자 및 언어재활사의 인식 조사 (2차)-1

연구 목적

본 설문은 아동들을 대상으로 메타버스를 활용하여 언어재활을 실시할 때, 양육자들과 언어재활사들이 생각하는 주요 고려 요인들을 분석하는데 목적이 있습니다.

연구 절차

회차	내용
1차 선택형 설문	메타버스를 활용한 언어재활에 대해서 질문에 따라 중요하다고 생각되는 항목들에 체크(v)해주시는 설문입니다.
2차 쌍대비교 설문	메타버스를 활용한 언어재활에 대해 제시된 비교의 속성에 따라 더 중요하다고 생각되는 속성 쪽의 숫자에 체크해 주시는 설문입니다.

이번 설문은 동일 주제에 대하여 양육자들과 언어재활사들에게 실시한 사전 조사 결과를 분석하여 선정된 요인들을 중심으로, 요인들간의 상대적인 중요도에 대한 응답자들의 인식을 조사하기 위한 것입니다.

연구 방법

0~12세 아동의 양육자 및 현재 현장에서 근무하고 있는 언어재활사를 대상으로 설문을 진행합니다. 아래 해당하는 QR 코드를 통해 온라인 설문지로 접속해주세요.
가능한 6월 30일까지 설문을 완료해주시면 감사하겠습니다.

☑ 아래 중 해당되는 설문에만 참여해주시길 바랍니다.

양육자

언어재활사

임의로 체크하실 경우 설문이 완료되지 않을 수 있으니 유의하시길 바랍니다.

나사렛대학교 일반대학원 언어치료학과
김하림 / 김정은 / 이진수

담당자 이메일 slpharim01@hanmail.net
지도교수 홍경훈

메타버스를 활용한 언어재활에 대한 양육자 및 언어재활사의 인식 조사 (2차)-2

메타버스(Metaverse)란?

현실을 디지털 기반의 가상 세계로 확장해 가상 공간에서 사회.경제.문화적 활동을 할 수 있게 만드는 시스템으로, 인공 가상 세계의 필수 기능을 갖춘 차세대 3차원(3D) 디지털 소셜 플랫폼 입니다.
위와 같이 현실을 디지털 기반의 가상현실로 확장한 시스템을 이용한 모든것을 메타버스라고 합니다. 본 연구에서는 메타버스를 플랫폼 안에서 정보를 교환하거나 직접 조작할 수 있는 것으로 정의하였습니다.

	증강현실 (Augmented Reality)	라이프로깅 (Life-logging)	거울세계 (Mirror worlds)	가상세계 (Virtual worlds)
정의	현실 공간에 가상의 2D 또는 3D 물체가 겹쳐져 상호작용하는 환경	사물&사람에 대한 일상적인 경험과 정보를 저장, 전송하는 기술	실제세계를 그대로 투영한 확장된 가상세계	디지털 데이터로 구축한 가상세계
구현가치	현실세계와 판타지, 편의성을 결합한 몰입 컨텐츠 제공	언제든지 확인 가능하고 타인과 공유할 수 있는 경험과 정보	외부정보를 가상공간에 통합, 확장	다양한 활동이 가능한 새로운 가상세계 제공
주요 서비스 사례	포켓몬GO(닌텐도) SNOW(네이버) AR HUD	S Health(삼성) 나이키+(나이키) SNS(인스타그램)	구글어스(구글) 네이버 지도(네이버)	포트나이트(Epic) 마인크래프트(MS) 제페토(네이버)
부작용 (도전요소)	현실이 중첩된 증강현실 공간 속의 혼란	초상권 및 재산권 침해 내부기밀 유출 및 겸업 금지 위반 등	정보 조작의 문제 거대 플랫폼 각인효과로 불공정거래	현실세계의 회피 도덕적 윤리적 문제를 일으킬 무질서
	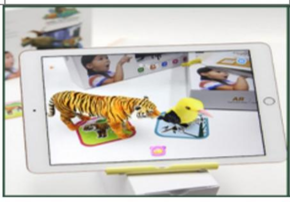	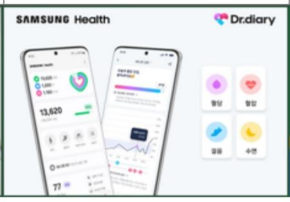	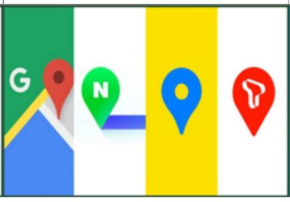	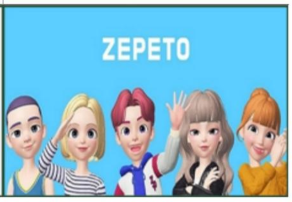

나사렛대학교 일반대학원 언어치료학과
김하림 / 김정은 / 이진수
담당자 이메일 slpharim01@hanmail.net
지도교수 홍경훈

본 설문은 "메타버스를 활용한 언어재활에 대한 양육자 및 언어재활사의 인식조사" 관련한 연구를 하기 위한 목적으로 만들어 진것입니다. 제공하신 정보는 학문적인 연구이외의 어떠한 목적으로도 사용하지 않을 것을 약속드립니다. 많은 협조 바랍니다.

본 설문은 귀하가 판단하는 메타버스를 활용한 언어재활 시 의사결정에 영향을 미치는 항목들의 상대적인 중요성에 대하여 작성하는 것입니다.

각 항목에 대한 설명을 잘 읽어보시고 양 쪽에 제시된 두 항목들 중에서 언어재활에 메타버스를 적용할 때 더 중요하다고 생각하는 항목 및 정도(수치)에 체크해주십시오.

※ 아래의 설문지 응답 예시 및 응답 결과에 대한 신뢰성 평가(응답 일관도) 관련한 사항을 주위 깊게 읽고 설문을 작성해 주시기 바랍니다.

A. 응답 예시

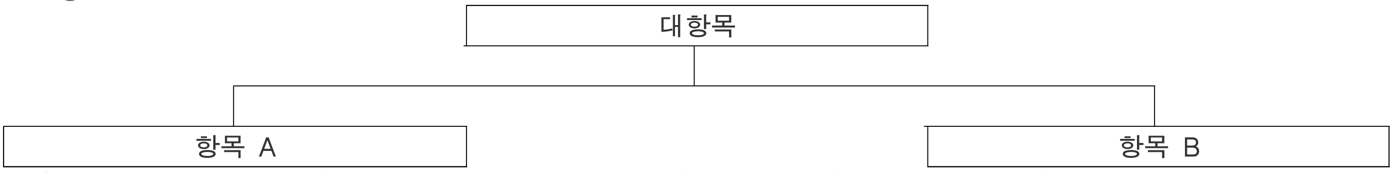

예} 상단의 대항목의 평가기준에서 판단할 때 항목 A가 항목 B보다 "매우 중요"하다고 생각하시면 아래와 같이 기입하면 됩니다.

항목	중요 ←																→ 중요	항목
	9	8	7	6	5	4	3	2	1	2	3	4	5	6	7	8	9	
항목 A																		항목 B

B. 응답 일관도

본 설문에서는 응답에 대한 신뢰도를 측정하기 위하여 일관도지수를 평가합니다. 일관도 지수가 0.10이상이 될 경우 응답결과를 신뢰할 수 없다고 판단되어 재설문하게 됩니다. 일관도 지수가 높게 나오는데는 크게 다음과 같이 두 가지 경우가 해당됩니다.

1. A > B : A가 B보다 2배 중요하다고 응답
2. A ≫ C : A가 C보다 4배 중요하다고 응답
→ [오류 1] B < C라고 응답, [오류 2] B ≫ C 라고 응답

[원인 1] 서수적 일관성 결여 : A > B > C의 순위가 바뀌게 응답
[원인 2] 기수적 일관성 결여 : 위 예에서 B가 C보다 9배 중요하다고 응답할 경우

다음부터는 귀하의 중요도 평가항목에 대한 본 설문입니다. 각 항목마다 질의에 맞게 판단하여 기입해 주시길 바랍니다.

1. 선택속성

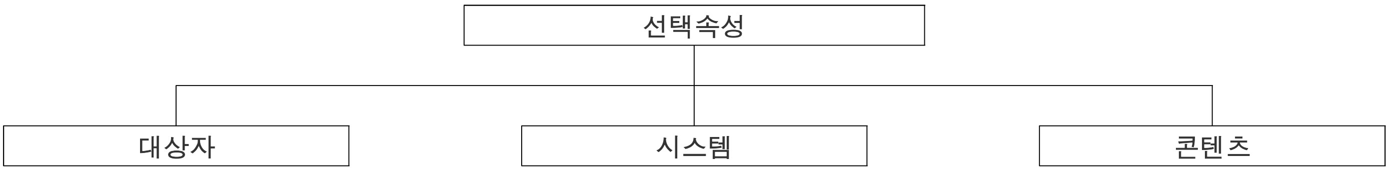

항목	항목 설명
대상자	메타버스 언어재활에 참여하는 대상자(언어재활을 제공하는 언어재활사, 제공받는 아동, 보조하는 양육자를 포함)
시스템	메타버스를 활용한 언어재활에 필요한 기기, 네트워크 및 공간 등의 시스템적 환경 전반
콘텐츠	메타버스를 활용한 언어재활에 필요한 콘텐츠의 양과 질적인 측면

▣ 선택속성 다음부터는 귀하의 중요도 평가항목에 대한 본 설문입니다. 각 항목마다 질의에 맞게 판단하여 기입해 주시길 바랍니다.

항목	중요 ◀																▶ 중요	항목
	9	8	7	6	5	4	3	2	1	2	3	4	5	6	7	8	9	
대상자																		시스템
대상자																		콘텐츠
시스템																		콘텐츠

2. 대상자

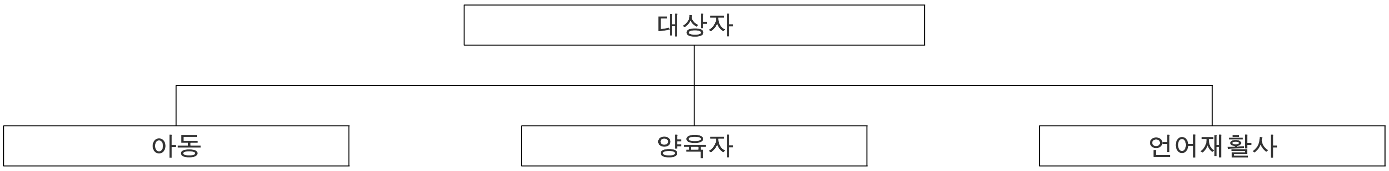

항목	항목 설명
아동	메타버스 언어재활을 제공받는 아동의 발달수준과 인지능력, 기기 및 콘텐츠에 대한 선호도
양육자	양육자의 메타버스 관련 기기 사용 능력과 선호도, 메타버스에 대한 인식, 보조자 역할
언어재활사	언어재활사의 메타버스 시스템 관리 능력, 언어재활 능력, 데이터에 대한 인식 및 관리 수준

▣ 대상자 다음부터는 귀하의 중요도 평가항목에 대한 본 설문입니다. 각 항목마다 질의에 맞게 판단하여 기입해 주시길 바랍니다.

항목	중요 ◀																▶ 중요	항목
	9	8	7	6	5	4	3	2	1	2	3	4	5	6	7	8	9	
아동																		양육자
아동																		언어재활사
양육자																		언어재활사

3. 시스템

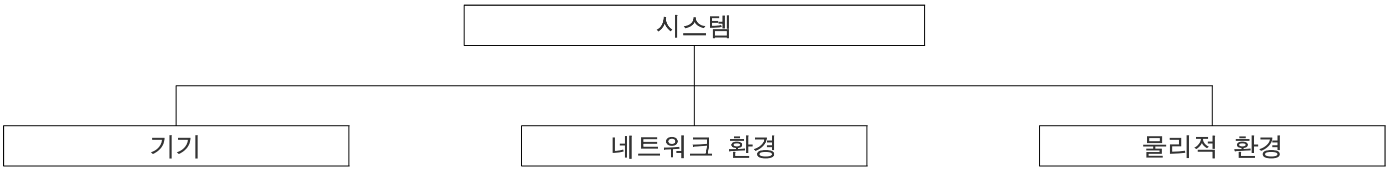

항목	항목 설명
기기	메타버스 기기의 안전성, 조작 용이성, 구매 및 유지보수 비용
네트워크 환경	메타버스 사용 시 네트워크의 연결성, 신속 적절한 대체 시스템 마련, 안정한 개인정보 보호 시스템 구축
물리적 환경	메타버스 언어재활을 위한 안전하고 독립적이고 조용한 공간

▣ 시스템 다음부터는 귀하의 중요도 평가항목에 대한 본 설문입니다. 각 항목마다 질의에 맞게 판단하여 기입해 주시길 바랍니다.

항목	중요 ◀																▶ 중요	항목
	9	8	7	6	5	4	3	2	1	2	3	4	5	6	7	8	9	
기기																		네트워크 환경
기기																		물리적 환경
네트워크 환경																		물리적 환경

4. 콘텐츠

- 콘텐츠
  - 콘텐츠의 양
  - 콘텐츠의 질

항목	항목 설명
콘텐츠의 양	메타버스 언어재활을 위한 콘텐츠의 다양성, 소요시간, 총량
콘텐츠의 질	메타버스 언어재활의 일반화 용이성, 흥미와 주의집중 유도, 의사소통적 상호작용 촉진

▣ 콘텐츠 다음부터는 귀하의 중요도 평가항목에 대한 본 설문입니다. 각 항목마다 질의에 맞게 판단하여 기입해 주시길 바랍니다.

항목	중요 ◀																▶ 중요	항목
	9	8	7	6	5	4	3	2	1	2	3	4	5	6	7	8	9	
콘텐츠의 양																		콘텐츠의 질

5. 아동

- 아동
  - 연령 및 신체 운동발달수준
  - 인지능력
  - 기기 및 콘텐츠에 대한 선호도

항목	항목 설명
연령 및 신체, 운동 발달수준	아동의 생활 연령과 메타버스 기기 사용을 위한 신체, 운동, 감각 발달 수준
인지능력	아동이 메타버스 콘텐츠를 활용할 수 있는 기초적 인지, 언어이해 및 주의집중 능력
기기 및 콘텐츠에 대한 선호도	메타버스 적용을 위해 필요한 기기(예. 컴퓨터, 스마트폰, 태블릿PC, VR기기 등)와 메타버스 콘텐츠에 대한 아동의 선호도

▣ 아동 다음부터는 귀하의 중요도 평가항목에 대한 본 설문입니다. 각 항목마다 질의에 맞게 판단하여 기입해 주시길 바랍니다.

항목	중요 ◀																▶ 중요	항목
	9	8	7	6	5	4	3	2	1	2	3	4	5	6	7	8	9	
연령 및 신체, 운동 발달수준																		인지능력
연령 및 신체, 운동 발달수준																		기기 및 콘텐츠에 대한 선호도
인지능력																		기기 및 콘텐츠에 대한 선호도

6. 양육자

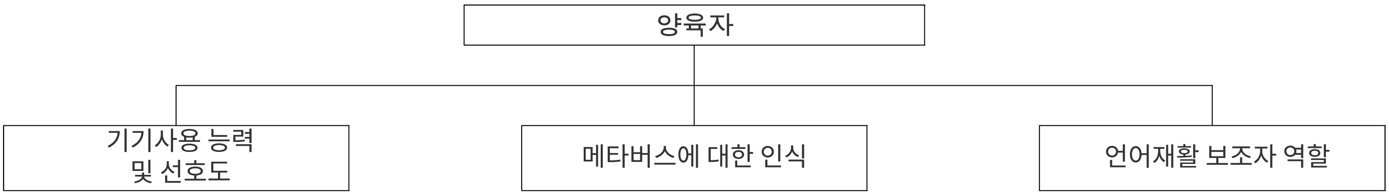

항목	항목 설명
기기사용 능력 및 선호도	메타버스 적용을 위한 기기(예, 컴퓨터, 스마트폰, 태블릿, VR 기기등)와 메타버스 콘텐츠에 대한 양육자의 선호도
메타버스에 대한 인식	메타버스를 활용한 언어 재활에 대한 양육자의 긍정적 인식 및 태도
언어재활 보조자 역할	메타버스 언어재활 중 양육자의 아동 관리 능력, 언어재활 과정 참여 및 지원

▣ 양육자 다음부터는 귀하의 중요도 평가항목에 대한 본 설문입니다. 각 항목마다 질의에 맞게 판단하여 기입해 주시길 바랍니다.

항목	중요 ◀															▶ 중요	항목
	9	8	7	6	5	4	3	2	1	2	3	4	5	6	7	8	9	
기기사용 능력 및 선호도																		메타버스에 대한 인식
기기사용 능력 및 선호도																		언어재활 보조자 역할
메타버스에 대한 인식																		언어재활 보조자 역할

7. 언어재활사

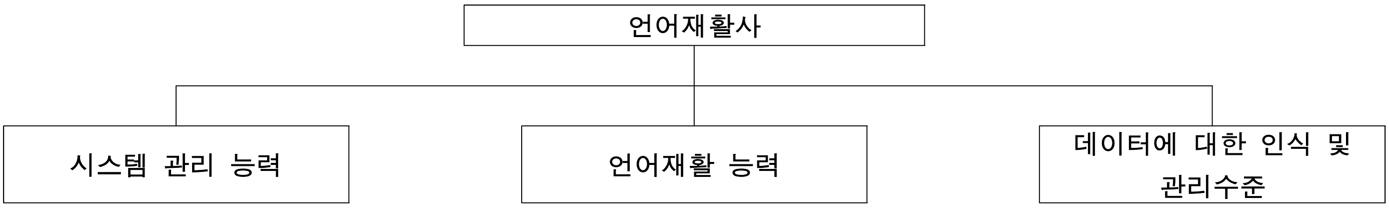

항목	항목 설명
시스템 관리 능력	메타버스 관련 기기 조작, 네트워크 연결 및 관리, 콘텐츠 사용 능력
언어재활 능력	메타버스를 활용한 언어재활 목표 설정 및 중재 실행
데이터에 대한 인식 및 관리수준	아동, 양육자의 개인 정보 보호를 위한 안전한 데이터 보호 체계 구축

▣ 언어재활사 다음부터는 귀하의 중요도 평가항목에 대한 본 설문입니다. 각 항목마다 질의에 맞게 판단하여 기입해 주시길 바랍니다.

항목	중요 ◀																▶ 중요	항목
	9	8	7	6	5	4	3	2	1	2	3	4	5	6	7	8	9	
시스템 관리 능력																		언어재활 능력
시스템 관리 능력																		데이터에 대한 인식 및 관리수준
언어재활 능력																		데이터에 대한 인식 및 관리수준

8. 기기

기기

- **기기 안전성**
- **조작 용이성**
- **기기 구매 및 유지보수 비용**

항목	항목 설명
기기 안전성	메타버스 활용 시 발생될 수 있는 신체적(예, 시청각적 자극, 넘어짐, 멀미 유발 등) 및 감정적(예, 놀람, 공포 등 유발) 문제에 대한 고려
조작 용이성	사용자 친화적 화면구성, 직관적 조작법으로 누구나 쉽게 다룰 수 있는 기기
기기 구매 및 유지보수 비용	양질의 메타버스 콘텐츠를 안정적으로 구현할 수 있는 사양의 기기 구매/유지보수 비용 수준

▣ 기기 다음부터는 귀하의 중요도 평가항목에 대한 본 설문입니다. 각 항목마다 질의에 맞게 판단하여 기입해 주시길 바랍니다.

항목	중요 ◀																▶ 중요	항목
	9	8	7	6	5	4	3	2	1	2	3	4	5	6	7	8	9	
기기 안전성																		조작 용이성
기기 안전성																		기기 구매 및 유지보수 비용
조작 용이성																		기기 구매 및 유지보수 비용

9. 네트워크 환경

네트워크 환경

- **네트워크 속도 안정성**
- **신속, 적절한 대체시스템 마련 가능성**
- **안전한 개인정보 보호 시스템 구축**

항목	항목 설명
네트워크 속도 안정성	메타버스 콘텐츠가 끊김 없이 빠르고 안정적으로 유지될 수 있는 네트워크 환경 고려
신속, 적절한 대체시스템 마련 가능성	기술적 문제, 네트워크 불안정 등 문제 발생 시 신속한 대체 시스템 마련
안전한 개인정보 보호 시스템 구축	아동, 양육자의 개인 정보 보호를 위한 안전한 데이터 보호 체계 구축

▣ 네트워크 환경 다음부터는 귀하의 중요도 평가항목에 대한 본 설문입니다. 각 항목마다 질의에 맞게 판단하여 기입해 주시길 바랍니다.

항목	중요 ◀																▶ 중요	항목
	9	8	7	6	5	4	3	2	1	2	3	4	5	6	7	8	9	
네트워크 속도 안정성																		신속, 적절한 대체시스템 마련 가능성
네트워크 속도 안정성																		안전한 개인정보 보호 시스템 구축
신속, 적절한 대체 시스템 마련 가능성																		안전한 개인정보 보호 시스템 구축

10. 물리적 환경

물리적 환경

안전한 공간 | **독립된 공간** | **조용한 공간**

항목	항목 설명
안전한 공간	움직임이 필요한 메타버스 활동 시 안전사고를 예방할 수 있는 공간 확보
독립된 공간	원활한 메타버스 적용과 대상자 개인정보의 외부 노출 차단을 위한 격리된 독립적 공간 확보
조용한 공간	원활한 메타버스 활동을 위하여 외부 소음 등의 방해 요인을 차단할 수 있는 조용한 공간 확보

▣ 물리적 환경 다음부터는 귀하의 중요도 평가항목에 대한 본 설문입니다. 각 항목마다 질의에 맞게 판단하여 기입해 주시길 바랍니다.

항목	중요 ◀																▶ 중요	항목
	9	8	7	6	5	4	3	2	1	2	3	4	5	6	7	8	9	
안전한 공간																		독립된 공간
안전한 공간																		조용한 공간
독립된 공간																		조용한 공간

11. 콘텐츠의 양

콘텐츠의 양

콘텐츠의 다양성 | **콘텐츠 소요시간** | **콘텐츠 총량**

항목	항목 설명
콘텐츠의 다양성	상황, 연령, 난이도에 따른 콘텐츠 다양성 확보
콘텐츠 소요시간	언어재활 목표를 달성할 수 있는 충분한 시간이 확보된 콘텐츠 확보
콘텐츠 총량	언어재활 활동의 지속성을 담보할 수 있는 충분한 콘텐츠의 총량 확보

▣ 콘텐츠의 양 다음부터는 귀하의 중요도 평가항목에 대한 본 설문입니다. 각 항목마다 질의에 맞게 판단하여 기입해 주시길 바랍니다.

항목	중요 ◀																▶ 중요	항목
	9	8	7	6	5	4	3	2	1	2	3	4	5	6	7	8	9	
콘텐츠의 다양성																		콘텐츠 소요시간
콘텐츠의 다양성																		콘텐츠 총량
콘텐츠 소요시간																		콘텐츠 총량

12. 콘텐츠의 질

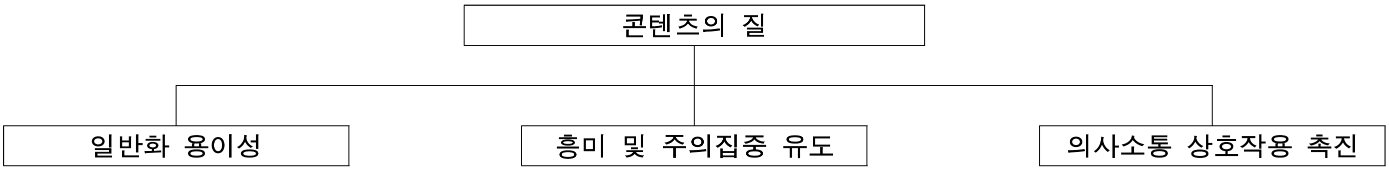

항목	항목 설명
일반화 용이성	일상생활과 유사한 가상 현실 내용으로 일상생활로 전이/일반화의 용이성 고려
흥미 및 주의집중 유도	아동의 참여동기 증진과 및 주의집중 유지를 유도할 수 있는 재미, 흥미 유발 고려
의사소통적 상호작용 촉진	아동과 콘텐츠 내 가상인물, 아동과 언어재활사 간의 의사소통적 상호작용을 촉진하는 쌍방향적인 내용의 콘텐츠

▣ 콘텐츠의 질 다음부터는 귀하의 중요도 평가항목에 대한 본 설문입니다. 각 항목마다 질의에 맞게 판단하여 기입해 주시길 바랍니다.

항목	중요 ◀																▶ 중요	항목
	9	8	7	6	5	4	3	2	1	2	3	4	5	6	7	8	9	
일반화 용이성																		흥미 및 주의집중 유도
일반화 용이성																		의사소통적 상호작용 촉진
흥미 및 주의집중 유도																		의사소통적 상호작용 촉진

다음은 귀하의 일반적인 현황에 대한 설문입니다. 각 항목마다 질의에 맞게 기입해 주시길 바랍니다.

---

1. 귀하의 성별은 무엇입니까?

☐ 남자 ☐ 여자

2. 귀하의 연령대는 무엇입니까?

☐ 20세 미만 ☐ 20~24세 ☐ 25~29세 ☐ 30~34세 ☐ 35~39세 ☐ 40~44세 ☐ 45~49세

3. 귀하의 학력은 무엇입니까?

☐ 고졸 미만 ☐ 고졸 ☐ 대학재학 ☐ 대학졸업 ☐ 대학원이상

4. 거주하시는 지역을 선택해 주세요.

☐ 서울시 ☐ 부산시 ☐ 대구시 ☐ 인천시 ☐ 광주시 ☐ 대전시 ☐ 울산시 ☐ 경기도 ☐ 강원도
☐ 충청북도 ☐ 충청남도 ☐ 전라북도 ☐ 전라남도 ☐ 경상북도 ☐ 경상남도 ☐ 제주도

5. 아동과의 관계를 선택해 주세요.

☐ 엄마 ☐ 아빠

6. 아동의 성별을 선택해 주세요.

☐ 남자 ☐ 여자

7. 아동의 연령을 선택해 주세요.

☐ 만 0~3세 ☐ 만 4~6세 ☐ 만 7~9세(초1~초3) ☐ 만 10~12세(초4~초6)

8. 아동이 해당하는 장애유형을 선택해 주세요.

☐ 장애 진단 없음 ☐ 단순 언어발달 지연 ☐ 지적장애 ☐ 자폐 범주성 장애 ☐ 청각장애
☐ 말소리장애

9. 기프티콘(커피)을 제공받으실 전화번호를 기재해 주세요.

[ ]

수고하셨습니다. 설문전송 버튼을 눌러 주십시오. [설문 전송] [다시 작성]

3. Stage 3 questionnaire, SLP version — demographic items only

Korean original. Items 1 to 4 and the final item were identical to the caregiver version (page 12) and are not repeated.

다음은 귀하의 일반적인 현황에 대한 설문입니다. 각 항목마다 질의에 맞게 기입해 주시길 바랍니다.

5. 귀하의 임상경력을 선택해 주세요.
   □ 1년 미만 □ 1~3년 □ 4~6년 □ 7~9년 □ 10년 이상

6. 임상에서 자주 접하는 대상자의 연령을 선택해 주세요.
   □ 만 0~3세 □ 만 4~6세 □ 만 7~9세(초1~초3) □ 만 10~12세(초4~초6)

7. 임상에서 주로 접하는 대상자의 장애 유형을 선택해 주세요.
   □ 장애 진단 없음 □ 단순 언어발달 지연 □ 지적장애 □ 자폐 범주성 장애 □ 청각장애
   □ 말소리장애

4. English translation

4.1 Explanatory material on the metaverse

The metaverse is a system that extends reality into a digitally based virtual world, allowing social, economic, and cultural activity to take place in a virtual space. It is a next-generation three-dimensional digital social platform with the essential functions of an artificial virtual world. Anything that uses a system extending reality into digitally based virtual reality in this way is termed a metaverse. In this study the metaverse is defined as an environment in which information can be exchanged or directly manipulated within the platform.

The material then set out four types — augmented reality, life-logging, mirror worlds, and virtual worlds — giving for each a definition, the value it delivers, representative commercial services, and known drawbacks, with illustrative images. Augmented reality was defined as an environment in which virtual 2D or 3D objects are superimposed on physical space and can be interacted with; life-logging as technology that stores and transmits everyday experience and information about objects and people; mirror worlds as an extended virtual world that mirrors the real world as it is; and virtual worlds as a virtual world built from digital data.

4.2 Questionnaire instructions

This questionnaire was prepared for the study "Caregiver and speech-language pathologist perceptions of metaverse-based speech-language therapy." We undertake that the information you provide will not be used for any purpose other than academic research.

The questionnaire asks you to judge the relative importance of the factors that bear on decisions about metaverse-based speech-language therapy. Please read the description of each item carefully and, for each pair presented, mark the item you consider more important when applying the metaverse to speech-language therapy, together with the degree of that importance.

A. Worked example. If, against the criterion given at the head of the cluster, you consider item A "very much more important" than item B, mark the corresponding value. The scale runs 9-8-7-6-5-4-3-2-1-2-3-4-5-6-7-8-9, where the central value of 1 indicates that the two items are equally important.

B. Response consistency. A consistency index is computed to assess the reliability of the responses. If the index exceeds the criterion, the responses are judged unreliable and the respondent is asked to answer again. A high index arises in two ways. (Cause 1) Loss of ordinal consistency: the ordering A > B > C is reversed in a later judgment. (Cause 2) Loss of cardinal consistency: having judged A twice as important as B and four times as important as C, the respondent then judges B nine times as important as C.

As noted on the first page of this file, the printed sheet states the conventional criterion of 0.10; the threshold implemented in the platform was CR ≤ 0.20.

4.3 Clusters, items, and operational definitions

Each of the 12 clusters was presented with a partial hierarchy identifying the elements to be compared, a table giving the operational definition of every element, and the pairwise-comparison items themselves. Clusters of three elements required three comparisons and the cluster of two elements required one, giving 34 comparisons in total.

1. Selection attributes

Item	Operational definition
Subject	Those taking part in metaverse-based speech-language therapy: the SLP who delivers it, the child who receives it, and the caregiver who assists.
System	The overall system environment required, including devices, network, and space.
Content	The quantity and quality of the content required.

2. Subject

Item	Operational definition
Child	The developmental level, cognitive ability, and device and content preferences of the child receiving therapy.
Caregiver	The caregiver’s device proficiency and preferences, awareness of the metaverse, and role as an assistant.
SLP	The SLP’s system management skills, ability to use the metaverse in intervention, and data awareness and management level.

3. System

Item	Operational definition
Device	Device safety, ease of use, and purchase and maintenance costs.
Network Connectivity	Network connectivity during use, provision of rapid and appropriate backup systems, and implementation of secure personal data protection.
Environment	A safe, private, and quiet space for metaverse-based therapy.

4. Content

Item	Operational definition
Amount of Content	The diversity, duration, and total volume of content.
Quality of Content	Generalization, induction of engagement and attention, and facilitation of communicative interactions.

5. Child

Item	Operational definition
Age and Physical-Motor Development Level	The child’s chronological age and the physical, motor, and sensory development required to use metaverse devices.
Cognitive Ability	The basic cognition, language comprehension, and attention needed to use metaverse content.
Device and Content Preferences	The child’s preferences regarding the devices required (e.g., computer, smartphone, tablet, VR headset) and the metaverse content.

6. Caregiver

Item	Operational definition
Device Proficiency and Preferences	The caregiver’s proficiency with and preferences regarding the devices required and the metaverse content.
Awareness of the Metaverse	The caregiver’s positive awareness of and attitude towards metaverse-based therapy.
Role as an Assistant in the Metaverse	The caregiver’s ability to manage the child during sessions and to participate in and support the therapy process.

7. SLP

Item	Operational definition
System Management Skills	Operating metaverse devices, establishing and managing the network connection, and using the content.
Ability to Use the Metaverse in Intervention	Setting therapy goals and delivering intervention using the metaverse.
Data Awareness and Management Level	Establishing a secure data protection system for the personal information of children and caregivers.

8. Device

Item	Operational definition
Device Safety	Consideration of physical risks (e.g., audiovisual stimulation, falls, motion sickness) and emotional risks (e.g., startle, fear) arising from use.
Ease of Use	Devices that anyone can handle easily, with a user-friendly interface and intuitive operation.
Device Purchase and Maintenance Costs	The cost of purchasing and maintaining devices of a specification able to run high-quality content reliably.

9. Network Connectivity

Item	Operational definition
Network Speed and Stability	A network environment in which content runs quickly and without interruption.
Rapid and Appropriate Backup Systems	Provision of a prompt alternative system when technical problems or network instability occur.
Implementation of Secure Personal Data Protection Systems	Establishing a secure data protection system for the personal information of children and caregivers.

10. Environment

Item	Operational definition
Safe Space	A space that prevents accidents during activities requiring movement.
Private Space	A separate space that supports smooth delivery and prevents external exposure of personal information.
Quiet Space	A quiet space that blocks external noise and other distractions.

11. Amount of Content

Item	Operational definition
Content Diversity	Content that varies by situation, age, and difficulty.
Content Duration	Content of sufficient duration to achieve therapy goals.
Total Content Volume	A total volume of content sufficient to sustain therapy over time.

12. Quality of Content

Item	Operational definition
Generalization	Virtual content resembling everyday life, supporting transfer and generalization to daily situations.
Induction of Engagement and Attention	Content that raises motivation to participate and sustains attention.
Facilitation of Communicative Interactions	Two-way content that promotes communicative interaction between the child and virtual characters, and between the child and the SLP.

4.4 Demographic items

Common to both versions

1. What is your gender? □ Male □ Female

2. What is your age band? □ Under 20 □ 20–24 □ 25–29 □ 30–34 □ 35–39 □ 40–44 □ 45–49

3. What is your highest level of education? □ Less than high school □ High school □ Enrolled in college □ College graduate □ Graduate degree or higher

4. Please select your region of residence. (17 metropolitan cities and provinces)

Final item. Please enter the telephone number to which the coffee voucher should be sent.

Caregiver version only

5. Please select your relationship to the child. □ Mother □ Father

6. Please select the child's gender. □ Male □ Female

7. Please select the child's age. □ 0–3 years □ 4–6 years □ 7–9 years (grades 1–3) □ 10–12 years (grades 4–6)

8. Please select the child's disability type. □ No disability diagnosis □ Developmental language disorder □ Intellectual disability □ Autism spectrum disorder □ Hearing impairment □ Speech sound disorder

SLP version only

5. Please select your years of clinical experience. □ Less than 1 □ 1–3 □ 4–6 □ 7–9 □ 10 or more

6. Please select the age band of the children you see most often in practice. □ 0–3 years □ 4–6 years □ 7–9 years (grades 1–3) □ 10–12 years (grades 4–6)

7. Please select the disability types you see most often in practice. □ No disability diagnosis □ Developmental language disorder □ Intellectual disability □ Autism spectrum disorder □ Hearing impairment □ Speech sound disorder
